# Rheological Considerations in Processing Self-Reinforced Thermoplastic Polymer Nanocomposites: A Review

**DOI:** 10.3390/polym14030637

**Published:** 2022-02-07

**Authors:** Mohamed Yousfi, Cédric Samuel, Jérémie Soulestin, Marie-France Lacrampe

**Affiliations:** 1 Univ Lyon, CNRS, UMR 5223, Ingénierie des Matériaux Polymères, Université Claude Bernard Lyon1, INSA Lyon, Université Jean Monnet, F-69621 Villeurbanne, France; 2 IMT Nord Europe, Institut Mines-Télécom, Univ. Lille, Centre for Materials and Processes, F-59000 Lille, France; cedric.samuel@imt-nord-europe.fr (C.S.); jeremie.soulestin@imt-nord-europe.fr (J.S.); marie-france.lacrampe@imt-nord-europe.fr (M.-F.L.)

**Keywords:** nanofibrillar-reinforced composites, polymer blends, in situ nanofibrillation, rheology

## Abstract

The present review relates to the field of nanocomposite materials comprising a thermoplastic nanofibrillar phase dispersed in a matrix that is also thermoplastic. The fact of forming the nanofibrillar phase in situ during melt processing gives it the role of a reinforcing nanofiller for thermoplastic materials. This paper discusses the major factors influencing the formation of self-reinforced nanofibrillar polymer composite (NFC) materials throughout manufacturing steps. More specifically, the rheological considerations allowing the prediction of the in situ nanofibrillation during melt blending and post-processing as well as the methods of production of these polymer nanocomposites are described. The major challenges related to the future development in the field of NFCs are addressed. The concept of self-reinforced nanofibrillar polymer materials shows great potential in lightweight eco-design processes and represents a new approach to polymer nanocomposite recycling for a variety of industrial applications.

## 1. Introduction

Nanocomposite materials comprising a polymer matrix in which reinforcing nanofillers are embedded to improve mechanical performance, such as silica, carbon and clay reinforcing elements, are well known. However, the reinforcing dispersed phase can also be made of polymer nanofibrils. In this case, one calls them nanofibrillar composites (NFCs). One of their advantages is that the reinforcing species is not present in the raw material, but comes into existence during blending of the compound and acts like “meltable solid fillers” [1,2]. Due to the fact that they are based only on thermoplastic polymers, their ability to be recycled is clearly improved compared with that of polymers strengthened by solid fibers (glass or carbon for example) [3,4]. Moreover, their density is lower (lightweight materials) and in certain configurations, they can present significantly superior mechanical and thermal properties [4,5]. They thus constitute a technically and economically interesting way to jointly address the issues related to the mechanical strengthening, lightening and recycling of thermoplastic polymers and nanocomposites.

The elaboration of NFCs involves mixing in the molten state a first polymer intended to form the matrix and a second polymer intended to form the nanofibrillar phase. The mixing conditions should be chosen to induce the formation of nanofibrils during cooling and drawing, followed by shaping through injection molding or extrusion at a temperature that is most often higher than the working temperature of the first matrix-forming polymer, but lower than that of the second polymer forming the nanofibrillar phase. The final shaping at a temperature below the processing temperature of the nanofibrillar phase allows the nanofibrils formed following the cooling of the polymer blends to be preserved. The orientation of the nanofibrils is mainly imparted during the shaping process of the polymer blends and to a lesser extent during the preparation of the molten resins. The nanofibrillar phase thus provides a reinforcement function thanks to an improvement in the mechanical resistance of the obtained nanocomposite materials [6,7]. During the manufacture of nanofibrillar composites (NFC) comprising a thermoplastic matrix (A) and a reinforcing thermoplastic component (B), the former generally has a melting or working temperature (TwA) below the corresponding temperature of component constituting the reinforcement, i.e., TwA < TwB.

In another configuration, when specific rheological conditions are met, the working temperature of polymer B (TwB), which forms the fibrillar phase, could be less than or equal to the melt processing temperature of polymer A, i.e., TwB < TwA. This arrangement renders possible an orientation of the fibrillar fibers during the melt shaping of the mixture of polymers A and B. The fact that this shaping takes place at a temperature greater than or equal to TwA, the end user of the polymer mixture is provided with more flexibility in the shaping thanks to the ductility of the material being adjusted according to the desired direction of stretching [2].

Advantageously, compared to traditional nanocomposites, the NFC materials exhibit an improvement in mechanical tensile strength and rigidity but this is often accompanied by an increase in ductility, and in particular in elongation at break, thus making the NFC composite material more ductile [8,9]. For the purposes of the present article, the term “nanofibrillar phase” includes any polymeric phase comprising nanofibrils, that is to say nanofibrils observable by an electron scanning microscope with nanosized diameters much smaller than their lengths and aspect ratios above 50 [10]. As in many immiscible polymer blends, one can observe large variations in dispersion homogeneity of the suspended polymer phase as well as in the strength of the matrix/nanofibrils interface, depending on a variety of factors: the thermal and rheological characteristics of neat polymers (melting temperatures, viscosity ratio, elasticity ratio, interfacial tension), the concentration of the dispersed phase and the conditions related to the processing (extrusion, stretching, injection molding, etc).

Most experimental studies have been devoted to the impact of the viscosity ratio on the formation of microfibers or nanofibrils and have often been carried out based on the Taylor theory, which takes into account the particular case of Newtonian droplets and matrix fluids. However, high molecular weight polymer blends under normal processing conditions exhibit non-Newtonian viscoelastic behavior and as a result the elasticity of the droplet and matrix phases has a significant impact on the fibrillation process under a flow field. Hence, a large section of the present paper is dedicated to these issues. Sufficient empirical and theoretical models giving detailed descriptions of the effect of interfacial tension in both shear and elongational flow are provided, followed by a presentation of the main elaboration and shaping processes of NFCs. Finally, the characteristic data describing their morphology and the experimental techniques used to determine them are addressed and this review ends by summarizing the major studies devoted to the investigation of material and process factors affecting the formation of the morphology and the resulting properties of NFCs.

## 2. From Polymer Blends to Nano-Fibrillar Polymer–Polymer Composites: Rheological Fundamentals

The scientific interest of self-reinforced polymer nanocomposites (NFCs) keeps increasing as these industrial materials gain in practical importance. The development surrounding them brings both economic and practical benefits, since they do not require expensive synthesis equipment nor protocols and because their properties are, potentially, easily and quickly adjustable to a large panel of targeted applications. Despite the fact that most polymer constituents of functional NFCs are immiscible, this apparent drawback can be seen as an opportunity to optimize their physical properties. This can be done by regulating dispersion properties such as the shape and average size of the reinforcing element as well as the size distribution of the dispersed phase, and the only requirement is a deep understanding of the rheological behavior of the blend components under real thermomechanical constraints imposed during the different stages of the melt processing.

When two immiscible polymers are processed together during melt mixing, an in situ morphology is developed. Factors impacting this morphology mainly include the components’ interfacial and rheological properties (i.e., the viscosity and elasticity of the molten materials), the blending conditions (i.e., the type of blending machine, the screw speed and time and temperature of blending) and the blend’s composition [11,12]. Nevertheless, there still exist contradictory results in the literature and there have yet to emerge any general rules making it possible to relate the aforementioned parameters to a blend’s final phase morphology. Moreover, the scientific understanding of how polymeric materials are dispersed remains unclear, thus limiting the technological capability of optimizing their properties.

Polymer blends can obtain a vast range of morphologies during melt processing and this is caused by the minor phase having a complex nature of deformation. Roughly, polymer blend morphologies can be grouped into two categories: systems that are either co-continuous or more dispersion-like. Research on the latter group has revealed a variety of morphologies, including spherical, ellipsoidal, fibrillar or droplet-in-droplet, even when identical compositions have been used. In particular, the droplet/fiber transition is a complicated procedure, both rheologically and thermally. In this case, and under particular processing conditions, the dispersed phase can create a fibrous structure, leading to one of the polymers either being reinforced or softened by the other.

The next section of the present review documents the conditions required to produce each type of blend system, in particular the rheological conditions of the polymer components that can result in a nano-fibrillar morphology, or in other words, droplet/fiber transition phenomena. The first studies carried out on polymer droplet deformation and breakup in immiscible blends were primarily devoted to systems where both the dispersed and continuous phases were purely viscous Newtonian liquids and thus did not display any quantifiable degree of elasticity. In such systems, the burst mechanism theory of the dispersed phase has been developed from a fundamental understanding of the mode of deformation and fracture of isolated Newtonian droplets in a purely viscous Newtonian matrix under well-defined conditions such as a simple shear flow field or an uniaxial extensional flow. The literature on the subject is rich and some excellent reviews include articles by Han [13]; Acrivos and Rallison [14,15]; Utracki [16]; and Elemans et al. [17,18,19].

Other studies, both experimental and theoretical, have dealt with non-Newtonian viscoelastic systems and have provided important insights by investigating viscoelastic polymer droplets in a Newtonian matrix or vice-versa, in a well-controlled shear field [20,21]. However, there are only a few attempts at exploring droplet deformation and fracture in polymer blends taking into account the effects of viscoelasticity of both the dispersed and suspending medium.

Therefore, in the following sections of this paper, we first give a brief overview on the miscibility of polymers after which we review the fundamentals of deformation and breakup, as well as the capillary instabilities in the case of Newtonian droplets suspended in a Newtonian matrix. In order to gain a deeper understanding of how polymeric droplets deform and burst while suspended in another polymer matrix, particular attention was paid to available information regarding non-Newtonian melt-mixed systems. We provide a state of the art detailing the theory of deformation in the case of viscoelastic droplets dispersed in a Newtonian surrounding matrix or Newtonian droplets suspended in a viscoelastic medium. Finally, the conditions to generate stable in situ nanofibrillar morphology in NFCs depending on the rheological characteristics of polymer components are highlighted.

## 3. Miscibility of Polymers

It is common knowledge that most pairs of polymers of a high molecular weight are immiscible and it is thermodynamically unfavorable for most polymeric materials to form homogeneous blends. To highlight the origin of this phenomenon, it is important to give some reminders on the thermodynamics of miscibility when blending polymers. The state of miscibility of any blend is governed by Equation (1), known as the Gibbs free energy of mixing, and defined according to

(1) $$\Delta G_{\mathrm{mix}}=\Delta H_{\mathrm{mix}}-T\;\Delta S_{\mathrm{mix}}$$

where $\Delta H_{\mathrm{mix}}$ and $\Delta S_{\mathrm{mix}}$ are respectively the enthalpy and the entropy of mixing. As stated by the second law of thermodynamics, the Gibbs free energy of mixing must be negative $\Delta G_{\mathrm{mix}}$ < 0 in order for the two components to mix. When two high molecular weight polymers are blended, the gain in entropy $\Delta S_{\mathrm{mix}}$ is negligible, and the free energy of mixing can be negative only if the same is true for the enthalpy of mixing $\Delta H_{\mathrm{mix}}$. The blending must thus be exothermic, which requires specific interactions between the components of the mixture. This is in turn is related to the chemical and physical structures of the materials and is characterized by the interaction parameter or the interfacial tension Γ of the system. Usually, only Van der Waals weak interactions occur in polymer blends, which explains why these systems are so often immiscible.

## 4. Summary of Droplet Deformation and Breakup Theories

### 4.1. Newtonian Fluids in Well-Defined Flow Fields

When immiscible fluids are blended in industrial processes, the morphology that is created is the product of droplet deformation, breakup and coalescence. Research on the breakup of Newtonian droplets and threads has been performed for quite some time already and the breakup of a viscous thread in a medium having no or negligible viscosity was described by Rayleigh [22] as early as in the 19th century. This theory was in the 1930s expanded by Tomotika [23] to include the breakup of viscous threads in an elongational flow, while during the same decade Taylor [24] explored how a liquid droplet deformed and broke up due to the motion of an immiscible, viscous suspending fluid. This lead to the publishing of a breakup criterion in both simple shear and hyperbolic flows. Thus, it seems that Taylor [24] was the first to have systematically investigated aqueous colloids, or in other words, how Newtonian droplets deform and break up at room temperature.

**Figure 1:**
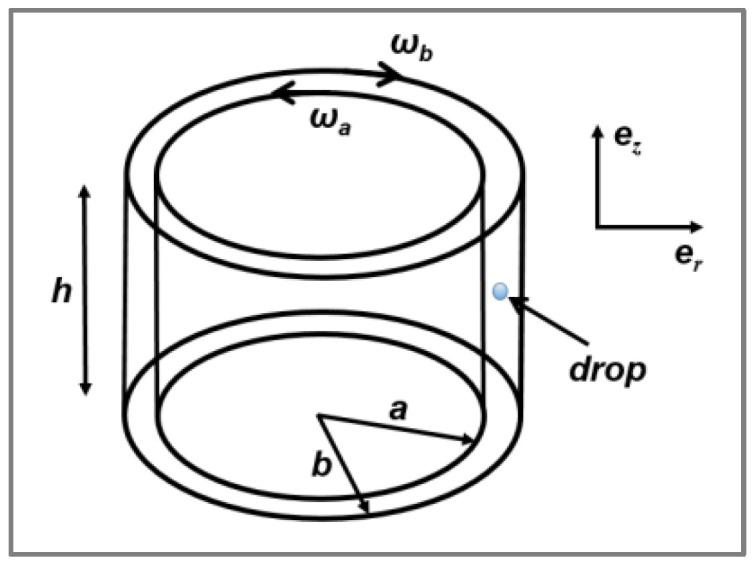
Scheme of the counter-rotating cylindrical Couette system. Adapted from reference [24].

Real mixing devices involve complicated flow fields, and since the deformation and break-up of droplets in such processes are more or less impossible to describe, most authors interpret the results they obtain, at least qualitatively, based on a description of particle break-up in simple flow fields. Taylor [24] carried out two basic experiments: he started by investigating the deformation of single Newtonian particles suspended in a second immiscible Newtonian continuous matrix under simple shear flow by means of a Couette type-apparatus (Figure 1) made up of two counter-rotating concentric cylinders. The speed of the two cylinders was adjusted to maintain a droplet in one position while being subjected to deformation at a shear rate of $\dot{\gamma }$.

**Figure 2:**
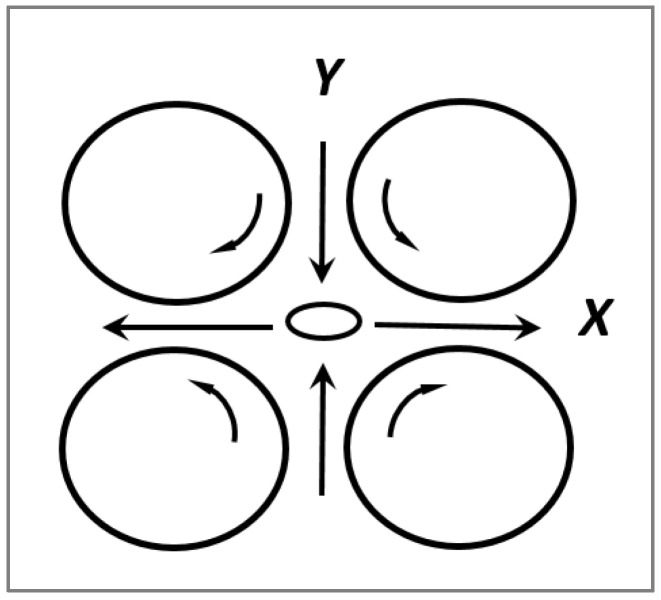
Schematic view of a four-roller mill apparatus. Adapted from reference [27].

He then built a four-roller mill apparatus to generate a hyperbolic flow so he could explore the mode of deformation and breakup under hyperbolic extensional flow (Figure 2). The experimental description of all devices used by Taylor has been detailed by Grace [25] and Levitt et al. [26].

Taylor found that two dimensionless parameters governed the deformation and breakup of particles in these purely viscous Newtonian systems: the ratio of the viscosity of the dispersed phase to that of its continuous counterpart, denoted k (k = η_d_/η_m_), and the Weber (or capillary) number, Ca, which is a dimensionless parameter representing the ratio of the viscous stresses exerted on the droplet by the external flow field to the interfacial tension forces restoring the particle to a spherical shape. The capillary number is defined as

(2) $$\mathrm{Ca}=\eta_{m}\dot{\gamma }/(\Gamma /R)$$

where η_m_ is the viscosity of the continuous phase, $\dot{\gamma }$ is the shear rate, R is the radius of the droplet prior to deformation, and Γ is the interfacial tension between the particle phase and its continuous counterpart. At a specific shear rate ($\dot{\gamma }$), the viscous forces are greater than the interfacial one, causing the droplet to break up. The (Ca) corresponding to the critical shear rate (${\dot{\gamma }}_{c}$) is called the critical capillary number and is denoted by Ca(crit) with

(3) $$\mathrm{Ca}(\mathrm{crit})=\eta_{m}{\dot{\gamma }}_{c}/(\Gamma /R)$$

**Figure 3:**
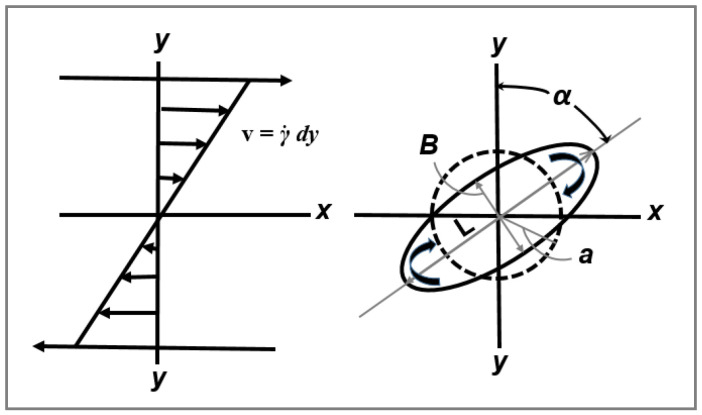
Definition of length (L), width (B), and orientation angle (α) of a deformed spherical droplet with an initial radius ‘a’ in simple shear flow. Adapted from reference [27].

Thus, in the absence of coalescence, the minimum obtainable droplet diameter in Newtonian melt-mixed blend systems (Taylor limit) can be estimated from Ca(crit). Taylor demonstrated that for small deformations in a rotational shear field, an initial spherical particle with a radius ‘a’ becomes deformed to an ellipsoidal shape with length L and width B (Figure 3). He defined the parameter Def, expressed as a function of the capillary number and the viscosity ratio k, according to:

(4) $$\mathrm{Def}=\frac{L-B}{L+B}=\mathrm{Ca}\;\cdot \;f(k)$$

(5) $$f(k)=\frac{19k+16}{16k+16}\;\mathrm{and}\;\alpha =\pi /4\;\mathrm{when}\;0\;<\;k\;\leq \;1\;(\mathrm{low}\;\mathrm{viscosity}\;\mathrm{ratio})\;$$

(6) $$f(k)=\frac{5}{4k}\;\mathrm{and}\;\alpha =\pi /2\;\mathrm{when}\;k\;>>\;1\;(\mathrm{limiting}\;\mathrm{deformation}\;\mathrm{without}\;\mathrm{burst})$$

Rumscheidt and Mason [28] were able to experimentally demonstrate that the deformation at burst (Def_burst_), under steady-state shearing flows (i.e., flows with a gradually increased shear rate and negligible inertia), is equal to the critical capillary number Ca(crit) within a range of viscosity ratios 0.1 ≤ k ≤ 1 with Def_burst_ ≈ Ca(crit) ≈ 0.5. It should be noted that the term f(k) ranged from 1 to 1.187 as k is increased from 0 to ∞, and that Def = 0 for a sphere. For Def ≥ 0.5 or L ≥ 3B, the droplet broke up [16,25].

According to Taylor, the critical shear rate ${\dot{\gamma }}_{c}$ can be expressed as:

(7) $${\dot{\gamma }}_{c}=\frac{\Gamma }{2\eta_{m}R}\cdot \frac{16k+16}{19k+16}$$

and the critical capillary number as:

(8) $$\mathrm{Ca}(\mathrm{crit})=\frac{1}{2}\cdot \frac{16k+16}{19k+16}$$

Cox [29] extended Taylor’s relation to time dependent flows (exclusively small deformations) as:

(9) $$\mathrm{Def}=\frac{5\;\left(19k+16\right)}{4\;\left(k+1\right)\;\sqrt{({\left(19k\right)}^{2}+{\left(20/\mathrm{Ca}\right)}^{2}}}$$

This corresponds to Taylor’s solution in the limits k→∞ or Ca→0 and led to a derivation of an expression for the orientation angle of the deformed droplet α according to:

(10) $$\alpha =\frac{1}{4}\;\pi +\frac{1}{2}\;\arctan\left(\frac{19k}{20/\mathrm{Ca}}\right)$$

All the analyses summarized here consider the behavior of isolated particles in a continuous medium. In usual blending operations, the dispersed phase fraction is important and the coalescence is not negligible. The influence of this parameter on the deformation of a droplet was taken into account by Choi and Schowalter [30]. For moderately concentrated emulsions in simple shear flow, they obtained:

(11) $$\mathrm{Def}=\mathrm{Ca}\;\left(\frac{19k+16}{16k+16}\right)\left(1+\frac{5\left(5k+2\right)}{4\left(k+1\right)}\;\varphi \right)$$

for Ca << 1 and 0 < k ≤ 1, where φ is the dispersed phase fraction.

(12) $$\mathrm{Def}=\frac{5\left(19k+16\right)}{4\left(k+1\right)\;\sqrt{({\left(19k\right)}^{2}+{\left(20/\mathrm{Ca}\right)}^{2}}}\left(1+\frac{5\left(5k+2\right)}{4\left(k+1\right)}\;\varphi \right)$$

when Ca ≤ 1 and k >> 1.

By using a four roller apparatus to generate a stationary hyperbolic extensional strain rate ($\dot{\varepsilon }$), Taylor theoretically demonstrated that one can predict droplet deformation and break-up from the previously mentioned shear rate equations by replacing $\dot{\gamma }$ by 2 × $\dot{\varepsilon }$, thus indicating that an elongational strain rate of magnitude $\dot{\varepsilon }$ has the same effect as a shear rate of magnitude 2 × $\dot{\varepsilon }$. For small deformations (low capillary numbers), the droplet deformation is given by:

(13) $$\mathrm{Def}=\frac{L-B}{L+B}=2\;\cdot \;{\mathrm{Ca}}_{e}\;\cdot \;\frac{19k+16}{16k+16}$$

where

(14) $${\mathrm{Ca}}_{e}=\frac{\eta_{m}\;\dot{\varepsilon }\;R}{\Gamma }$$

At high capillary numbers (the interfacial tension is neglected), the deformation of drop and matrix are affine and the deformation

(15) $$\mathrm{Def}=\frac{5}{2k+3}$$

This equation predicts that highly viscous drops (k > l) undergo less deformation than their surrounding medium. As expected, for an iso-viscous drop and matrix (k = 1), the deformation is identical in the two phases. When the viscosity ratio is very small (k << 1), the droplet deforms more than the matrix, thereby giving

(16) $$\mathrm{Def}=\frac{5}{3}$$

The time evolution of the shape of a drop Def(t) that is suddenly subjected to a hyperbolic extensional flow rate $\dot{\varepsilon }$ is given by Cox [29]:

(17) $$\mathrm{Def}=2\;{\mathrm{Ca}}_{e}\;\frac{19k+16}{16k+16}\;\left(1-\exp\left(-\beta \;t\right)\right)\;\mathrm{with}\;\beta =\frac{20\;\dot{\varepsilon }\;}{19k\;{\mathrm{Ca}}_{e}}$$

**Figure 4:**
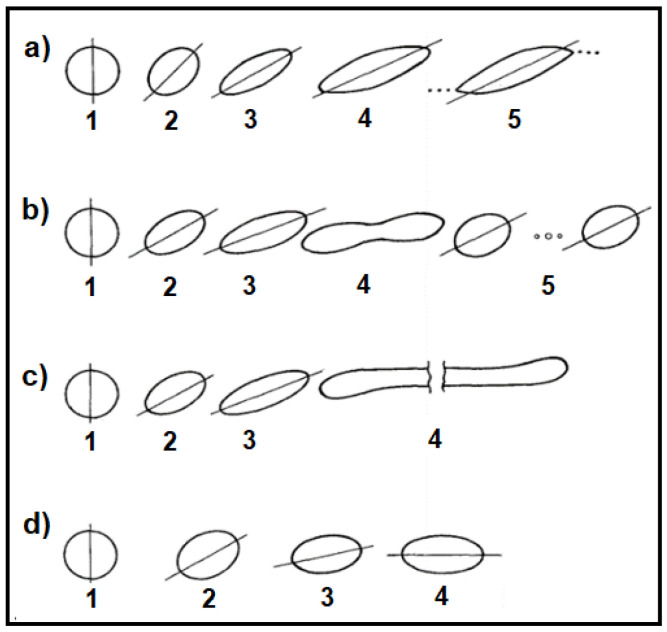
Various deformation modes of a particle subjected to a simple shear flow for (Ca ≥ Ca(crit)). (**a**) k = 2 × 10^−4^; (**b**) k = 1; (**c**) k = 0.7 and Ca = Ca(crit)); (**d**) k = 6. Adapted from [28].

Many other authors have studied the deformation and burst conditions of particles in a shear-field [31,32]. Rumscheidt and Mason [28], for instance, differentiated several categories of deformation and fracture of a drop in a simple shear flow according to the values of the viscosity ratio k and the capillary number Ca (Figure 4). In the case where no shear is applied, the droplet has a spherical shape (cf. photographs of drop number 1 in Figure 4), whereas under low deformation (Ca << Ca(crit)), deformation occurs and the particle takes the form of an ellipsoid (cf. photographs of drop number 2). However, for larger shear rates (Ca ≥ Ca(crit)), four types of drop fracture occur (cf. photographs of drops number 3, 4, 5…):

- Class (a): for k < 0.2; the particle takes on a sigmoidal shape and tiny drops break away from its ends (tip streaming phenomenon).
- Class (b): 0.2 ≤ k < 1; the particle’s central portion suddenly extends into a cylindrical shape, creating a neck in the middle (necking mechanism). The neck becomes progressively thinner until two identical daughter droplets and three satellite droplets are formed.
- Class (c): 1 ≤ k < 4; the droplet extends into a long thread that gets progressively longer until breaking up into a large number of fine particles.
- Class (d): k > 4; no burst occurs regardless of the applied shear rates. The particle deforms into an ellipsoid and orients along the flow without showing any signs of disintegration even at the upper limit of Ca of the apparatus ($\dot{\gamma }$ up to 40 s^−1^). This is predicted by $\mathrm{Def}=\frac{5}{2k+3}$, yielding Def < 0.3 for k > 4, which can be regarded as insufficient for breakup.

At a fixed viscosity ratio k with (1 ≤ k < 4), several modes of deformation and breakup are possible in a shear flow and depend on the capillary number:

- Ca < 0.1 Ca(crit): no droplet deformation occurs. The interfacial energy dominates.
- Ca(crit) ≤ Ca < Ca(crit): there is slight droplet deformation without break-up, and a stable form is reached.
- Ca(crit) ≤ Ca ≤ 2 Ca(crit): the interfacial stress is dominated by the viscous stress causing the droplet to become unstable and breakup to occur as a splitting of the particle into two equal parts before elongation into a filament can be achieved. The radius of the drops can be calculated as:
  (18) $$R_{\mathrm{drops}}=\frac{Ca\left(\mathrm{crit}\right)}{\eta_{m}\;\dot{\gamma }/\Gamma }\;2^{-1/3}$$
- Ca(crit) < Ca ≤ 4 Ca(crit): particle deformation occurs leading to a long, unstable fiber, followed by fragmentation through ‘end-pinching’ or Rayleigh capillary instabilities giving rise to a large number of smaller drops [22,23,33].
- Ca > 4 Ca(crit): the shear stress is much stonger than the interfacial stress, causing the droplets to be deformed into long fibrils that do not break, but rather rotate in the flow field. In this case, the formation of a stable fibrillar structure can be obtained under specific conditions [17,34,35,36] (see later).

**Figure 5:**
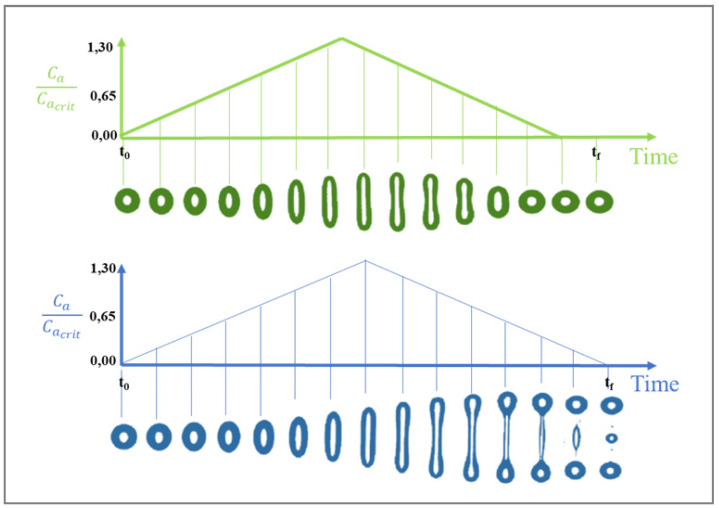
Droplet response to a triangular flow rate–time profile where the maximum capillary number was reached in 42 (**top**) and 46 (**bottom**) seconds. Adapted from [37].

All the aforementioned studies on droplet deformation and breakup in Newtonian systems were realized under quasi-equilibrium conditions, without including the time. In general, the flow field in industrial mixers is not homogeneous. As a result, the flow rate experienced by a moving droplet is time-dependent (transient flow) and the burst does not occur unless there is enough time provided for the breakup. An example of the influence of time-dependent flow on droplet deformation is given in Figure 5. The Figure displays two single drop experiments in an elongational flow generated by a device with opposed jets [37]. A polybutadiene droplet (η_d_ = 12 Pa·s) was used in a polydimethylsiloxane matrix (η_m_ = 9 Pa·s), and the radius of the droplet was 0.48 mm, whereas the interfacial tension was equal to 4 mN/m. In both experiments, the capillary number was first increased from zero to 1.3 Ca(crit) and then decreased back to zero. In the first experiment, the maximum capillary number reached 42 s after the start of the experiment: the droplet deformed as the capillary number increased and then retracted back to a sphere again. In the second experiment, the capillary number was increased at a slower rate and the maximum capillary number was reached in 46 s. In this case, the drop did not retract back to a sphere but continued to elongate until breakup occurred. It should be noted that the viscosity ratio k = 1.3 with (Ca/Ca(crit) < 2) in both experiments.

**Figure 6:**
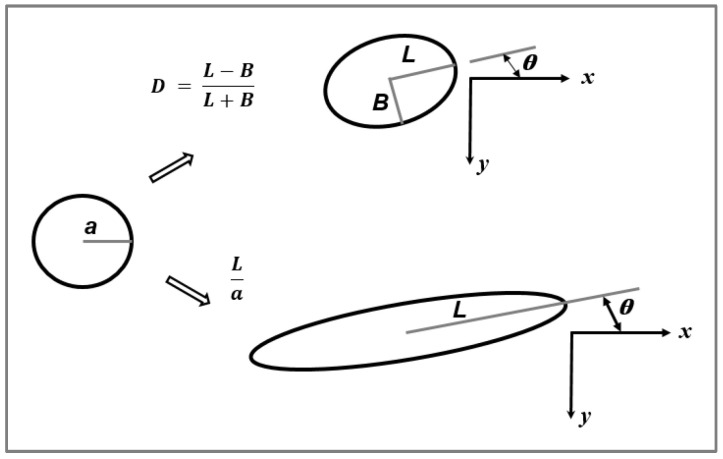
Scalar measures of deformation and orientation. Adapted from [38].

For a particle undergoing high deformation in a medium (reduced capillary number Ca/Ca(crit) > 1), breakup is caused by the significant stresses during flow. However, it has been seen that surface instabilities can also give rise to rupture. In particular, when Ca/Ca(crit) > 2, a long thread with L/a > 6 (where L is the length after deformation and ‘a’ is the initial particle diameter (cf. Figure 6), suspended in a different medium is in non-equilibrium, and its stability therefore depends on the flow conditions, the interfacial tension, its own rheological properties as well as those of the liquid it is suspended in [38]. Stone et al., [38] found that particle breakup follows one of two mechanisms depending on the elongation ratio (L/a). When (6 < L/a < 15), the particles broke up because of ‘end-pinching’ (i.e., the pinching off of the ends of a stretched drop from the central thread due to the particle relaxing after a sudden change in flow conditions). When (L/a > 15), capillary instabilities (known as Rayleigh disturbances) are the predominant breakup cause. One should keep in mind that the fact that polymers exhibit high viscosities and low surface tensions result in elevated relaxation times for the drop and consequently, breakup by end-pinching may not occur in such systems. This mechanism would, however, be the dominant mode of rupture for liquids with a low viscosity.

In 1879, Rayleigh was the first to treat capillary instabilities for a jet of a viscous fluid in air and his findings were later extended by Tomotika [23] to the case of a single cylindrical viscous thread embedded in a quiescent Newtonian medium (after the flow had been stopped).

**Figure 7:**
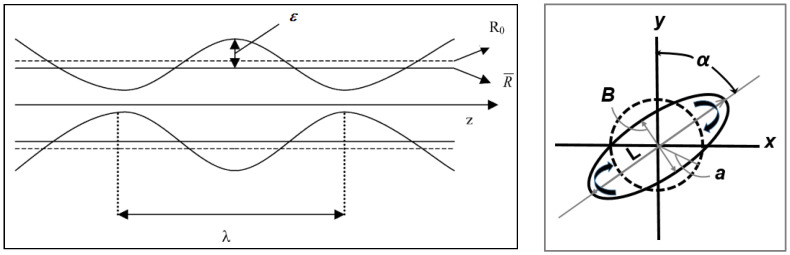
Left: schematic representation of capillary instability (sinusoidal distortion) taking place as a cylindrical thread with an initial radius R_0_ is extended. Here, $\bar{R}=\sqrt{R_{0}{}^{2}-\varepsilon^{2}/2}$ (Equation (19)) is the average radius, ε is the amplitude of the distortion, λ is the wavelength and z is the Cartesian coordinate along the cylinder’s main axis. Right: Length (L), width (B), and orientation angle (α) of a deformed spherical droplet with an initial radius ‘a’ in simple shear flow. Adapted from [39].

**Figure 8:**
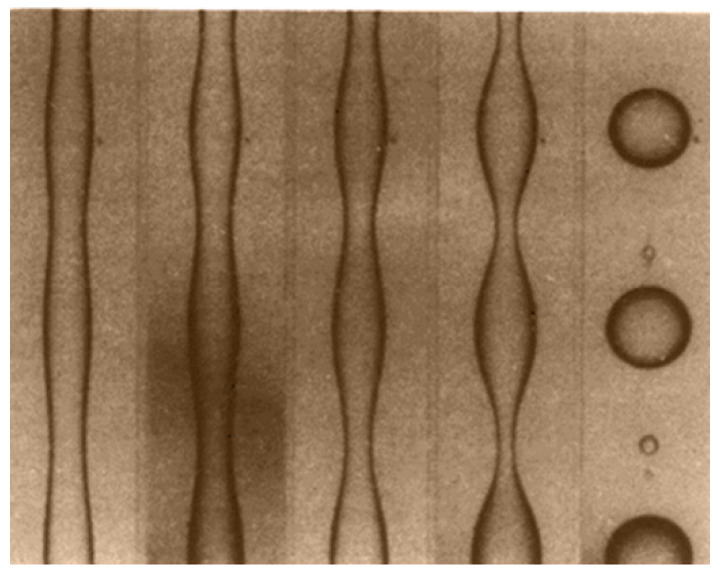
Sinusoidal distortions for a thread (diameter 55 µm) of polyamide 6 embedded in a PS matrix at 230 °C, adapted from [17]. The photographs were taken at: t = 0, 15, 30, 45, 60 s.

This theory assumes that under supercritical conditions Ca/Ca(crit) > 2, once the droplet has become highly extended (high L/B with B = 2 R_0_, see Figure 7), very small sinusoidal disturbances appear on the surface of the fibril see Figure 8. Distortions with a wavelength, λ, larger than the original circumference of the fibril, 2πR_0_, give rise to a reduction in interfacial surface area and as a result only these distortions are able to grow (see Figure 7).

A dimensionless wave number of distortion, X, is given by

X = 2πR_0_/λ (19)

where X varies between zero and unity. The distortion amplitude, ε, is assumed to increase exponentially with time, according to

(20) $$\varepsilon =\varepsilon_{0}\exp(qt)$$

where ε_0_ is the amplitude of the distortion at time t = 0. A lower limit of ε_0_ can be obtained by thermal fluctuations and was estimated by [40]:

(21) $$\varepsilon_{0}=\sqrt{\left(21\kappa_{B}T)/(8\pi^{3/2}\Gamma \right)}$$

where κ_B_ is the Boltzmann constant, T is the absolute temperature and Γ is the interfacial tension. According to [40], ε_0_ ≈ 10^−9^ m for Γ = 10 mN.m^−1^. Ref. [41] gaves a higher estimate of 10^−8^ to 10^−7^ m.

**Figure 9:**
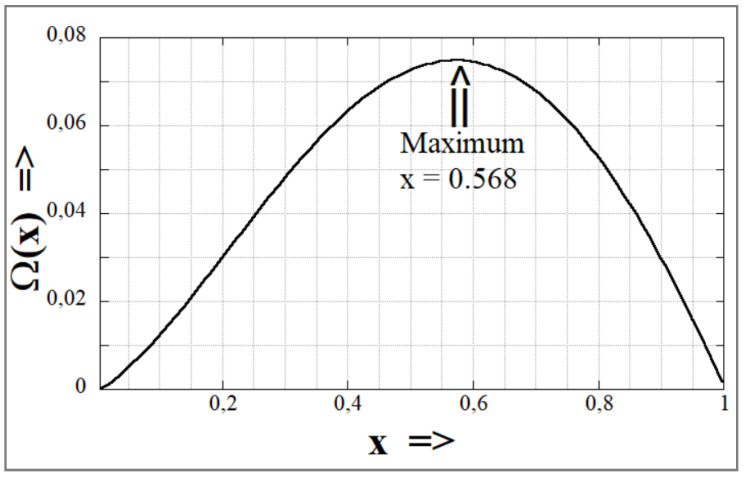
Curve of Ω(k, X) for a viscosity ratio k = 0.91. Adapted from [39].

The growth rate of the distortion (q) is expressed as

(22) $$q=\Gamma \Omega (k,X)/2\eta_{m}R_{0}$$

where η_m_ is the matrix viscosity, R_0_ is the initial radius of the thread (R_0_ = B/2; see Figure 7) and Ω(k, X) is a complex function of the characteristic wave number, X, of the perturbation and the viscosity ratio, k, of the system in question. When the function Ω(k, X) is at its maximum, breakup of the thread occurs. Values of Ω(k, X) can be calculated from Tomotika’s original equation [39] and Figure 9 shows the function Ω(k, X) for two immiscible liquids with a viscosity ratio k = 0.91. Tomotika observed that the breakup of the fiber took place at an unique value for the dominant wave number, X_m_ = 0.568 with a value of Ω(k, X_m_) = 0.074.

**Figure 10:**
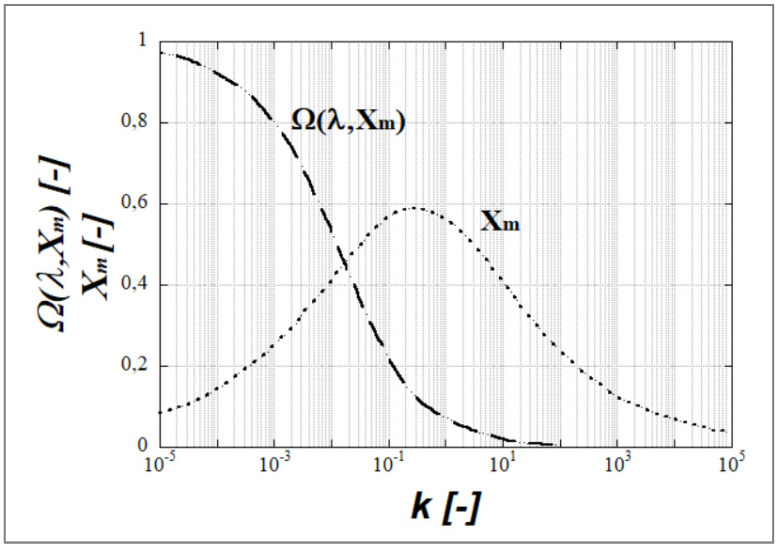
The wavenumber and growth rate of the dominant wavelength. Adapted from [20,42].

Figure 10 shows a plot of the values of the dominant growth rate Ω(k, X_m_) and the dominant wave number X_m_ vs. the viscosity ratio k [17,20,42]. For k→0, the dominant growth rate function Ω(k, X_m_) approached unity and for k ≈ 100, Ω(k, X_m_) it was equal to zero. For k ≈ 0.3, the dominant wavelength λ_m_ was at its minimum (the wave number X_m_ was maximal) indicating a maximum amount of capillary instabilities in this region of the viscosity ratio.

For 0.01 ≤ k ≤ 10, [16] used the following equation to fit the function Ω(k,λ_m_):

(23) $$\Omega (k,\lambda_{m})=\exp[b_{0}+b_{1}\log k+b_{2}{\left(\log k\right)}^{2}+b_{3}{\left(\log k\right)}^{3}+b_{4}{\left(\log k\right)}^{4}]$$

where b_0_ = −2.588, b_1_ = −1.154, b_2_ = 0.03987, b_3_ = 0.0889, and b_4_ = 0.01154.

Tomotika estimated that the breakup occurs when the amplitude of the deformation ε reached a critical value corresponding to the average radius of the thread where

(24) $$\varepsilon_{b}=\bar{R}=0.81\cdot R_{0}$$

and

(25) $${\bar{R}}^{2}=R_{0}{}^{2}-(\varepsilon^{2}/2)$$

according to the condition of conservation of volume (see Figure 6). The droplet size formed upon rupture of the fibril can be calculated from the following equation [19]:

(26) $$R_{\mathrm{at}\;\mathrm{breakup}\;\mathrm{of}\;\mathrm{the}\;\mathrm{fibril}}=R_{0}\sqrt[{3}]{\frac{3\pi }{2X_{m}}}\approx 2R_{0}$$

For example, if the viscosity ratio k = 0.91, X_m_ = 0.568, so that the diameter of the droplets formed after breakup of the fiber would be approximately twofold that of the original fiber.

The time required for the fiber to rupture can be used to evaluate the stability of a fibrillar structure.

For a thread suspended in a matrix, where both liquids are Newtonian, the breakup time in a shear field, t_b_, can be determined using the equation

(27) $$t_{b}=\frac{2\eta_{m}R_{0}}{\Gamma \Omega (k,\lambda_{m})}\ln\left(\frac{\varepsilon_{b}}{\varepsilon_{0}}\right)$$

or in a dimensionless form (reduced breakup time) as

(28) $$t_{b}{}^{*}=\frac{t_{b}\dot{\gamma }}{2\mathrm{Ca}}=\frac{2}{\Omega (k,\lambda_{m})}\ln\left(\frac{0.81R_{0}}{\varepsilon_{0}}\right)$$

When deformation of the fiber takes place during flow, there occurs a superposition of a supplementary flow-induced deformation on the one due to Rayleigh instabilities, resulting in the breakup time given by Equation (22) no longer being valid [43,44]. The fiber extension due to the flow reduces the instability growth rate whereby the fiber stabilizes. This assumes that (i) the fiber deforms affinely with the matrix (Ca/Ca(crit) ≥ 2); (ii) the fiber is ruptured when the instability-induced local diameter reduction rate is greater than that caused by the fluid deformation; and (iii) that the instability wavelength is independent of fluid flow [43].

The average diameter reduction rate due to the Rayleigh instability (dB/dt)_Rayleigh_ can be determined as the diameter of the fiber divided by the breakup time:

(29) $${\left(\frac{\mathrm{dB}}{\mathrm{dt}}\right)}_{{}_{\mathrm{Rayleigh}}}=\frac{-B}{t_{b}}=\frac{-\Gamma \Omega (k,\lambda_{m})}{\eta_{m}\ln(\varepsilon /\varepsilon_{0})}$$

In a simple shear flow with an affine deformation, if the conservation of volume condition is employed, a spherical droplet with an initial diameter ‘a’ bedomes deformed into a fibril with the smallest dimension B (see Figure 7b) according to [17]:

(30) $$B=a{\left(1+{\left(\dot{\gamma }t\right)}^{2}\right)}^{-1/4}$$

where ‘t’ is time, and $\dot{\gamma }$ is the shear rate. In the case where the fiber is highly elongated (near breakup),

(31) $$1+{\left(\dot{\gamma }t\right)}^{2}\approx {\left(\dot{\gamma }t\right)}^{2}$$

and the diameter reduction rate is expressed as:

(32) $${\left(\frac{\mathrm{dB}}{\mathrm{dt}}\right)}_{{}_{\mathrm{Shear}}}=\frac{-a}{2}{\dot{\gamma }}^{-1/2}t^{-3/2}$$

Since rupture is assumed when ${\left(\frac{\mathrm{dB}}{\mathrm{dt}}\right)}_{{}_{\mathrm{Shear}}}$ is equal to ${\left(\frac{\mathrm{dB}}{\mathrm{dt}}\right)}_{{}_{\mathrm{Rayleigh}}}$, breakup should take place when:

(33) $$\frac{\Gamma \Omega (k,\lambda_{m})}{\eta_{m}\ln(\varepsilon /\varepsilon_{0})}\geq \frac{a}{2}{\dot{\gamma }}^{-1/2}t^{-3/2}$$

which yields the following equation for breakup time:

(34) $$t_{b}={\left[\frac{\eta_{m}\ln(\varepsilon /\varepsilon_{0})}{\Gamma \Omega (k,\lambda_{m}){\dot{\gamma }}^{1/2}}\;\frac{a}{2}\right]}^{2/3}$$

Consequently, during shear, the breakup time is also a function of the initial particle diameter and of the shear rate. Since there is no theoretical value for the term ln(ε/ε_0_), it was evaluated by experimental results [43,44]. A value of 20 was found to give the best agreement between theory and experiment.

We can use the same analysis for extensional flow. In this case, the particle deformation is expressed as:

(35) $$B=a\;\exp(-{\dot{\varepsilon }}_{\mathrm{el}}\;t/2)$$

where ${\dot{\varepsilon }}_{\mathrm{el}}$ is the stretching rate [19]. The breakup time in elongational flow then becomes:

(36) $$t_{b}=\left(\frac{2}{{\dot{\varepsilon }}_{\mathrm{el}}}\right)\ln\left(\frac{\eta_{m}\ln(\varepsilon /\varepsilon_{0}){\dot{\varepsilon }}_{\mathrm{el}}}{\Gamma \Omega (k,\lambda_{m})\;}\frac{a}{2}\right)$$

**Figure 11:**
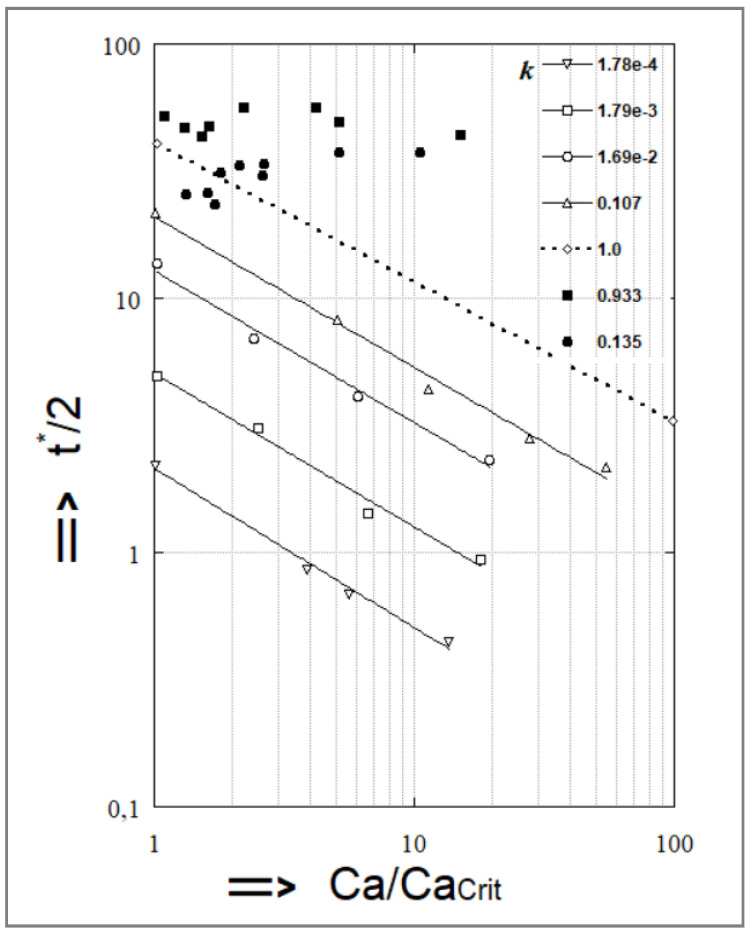
Effect of higher Ca/Ca(crit) values on the dimensionless burst time t_b_^*^ (from Grace [1982] and Elemans [1988]). ■: k= 0.0002; +: k = 0.0018; *: k = 0.0169; □: k = 0.107; x: k = 0.135 (Elemans); ▲: k = 0.933 (Elemans). The dashed line was obtained by plotting t_b_^*^ at Ca/Ca(crit) = 1 on the vertical axis and assuming an identical slope as for the solid lines. Reproduced from [17] with permission from Elsevier.

The deformation of Newtonian droplets into fibers and the time corresponding to their complete breakup under a shear field, (t_b_^*^), have been the subjects of several studies [17,38,42]. Elemans et al. [17] compared the results concerning the dimensionless time for breakup (t_b_^*^) with data from similar research by Grace [25] and Figure 11 summarizes the findings. Grace demonstrated that (t_b_^*^) became lower as Ca/Ca(crit) increased and the viscosity ratio k decreased. The following empirical formula was obtained from Figure 11:

(37) $$t_{b}{}^{*}=84\cdot k^{0.345}\cdot {\left[\left(\mathrm{Ca}/\mathrm{Ca}(\mathrm{crit})\right)\right]}^{-0.559}$$

The importance of this equation is the fact that the time required for particle rupture can be approximated from Ca/Ca(crit) and the viscosity ratio k through comparison with the average residence time of the fiber in the die (this value should be lower than the time of fiber breakup in order to obtain a stable fibrillar morphology). However, according to Figure 11, the time for breakup did not decrease as Ca/Ca(crit) increased in the case of Elemans experiments. He explained this difference with the fact that Grace possibly observed end-pinching, which indeed would give rise to much lower values for (t_b_^*^).

**Figure 12:**
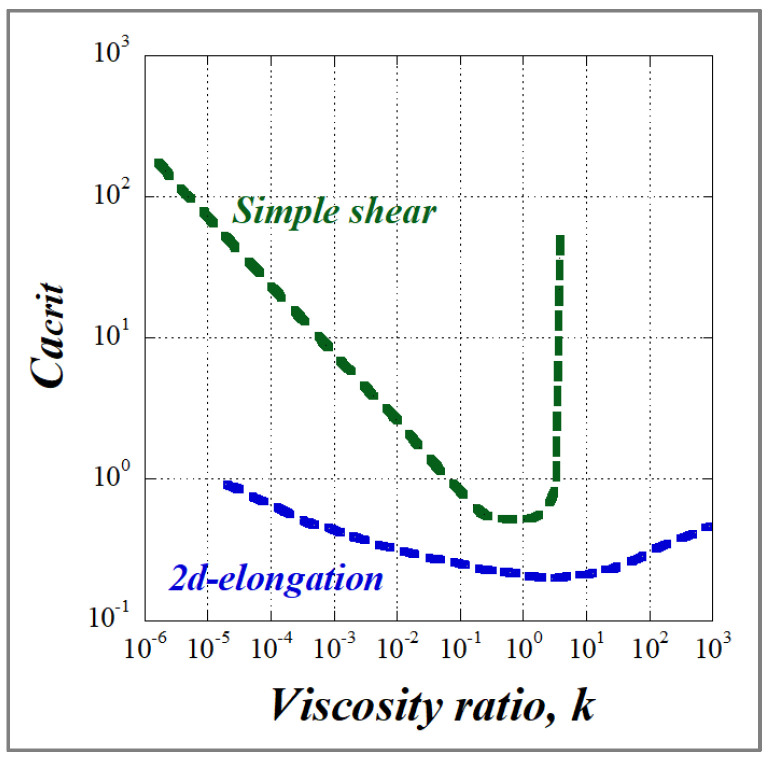
The critical capillary number Ca(crit) as a function of the viscosity ratio k (also known as the Grace curve) for the rupture of an initially spherical drop in quasi-steady homogeneous shear flow (under simple shear and a plane hyperbolic flow). Adapted from reference [25].

Experimental data on how the critical capillary number relates to the viscosity ratio in the case of Newtonian blends under shear and elongational flow have been reported by several authors [15,32,45,46,47,48], but the work by Grace [25] is probably the most cited. Figure 12 depicts the Grace curve which is one of the most used graphs in the field of dispersive mixing. It relates the critical capillary number Ca(crit) to the viscosity ratio k (k varies between 10^−6^ and 950) for the rupture of an initially spherical drop in a quasi-steady homogeneous flow. From Grace’s data one can conclude that the critical capillary number Ca(crit) depends both on the viscosity ratio k and on the flow type. Ca(crit) is, regardless of k, lower for a hyperbolic flow (2D elongation) than for a simple shear flow and it is thus easier to deform and rupture a particle under extensional flow than under shear flow.

Since a hyperbolic flow does not permit the rotation of drops, deformation occurs even for highly viscous droplets (k > 100). However, beyond k ≈ 4, the critical capillary number becomes very large, making it impossible to break a droplet even under simple shear due to the flow’s rotational character. Droplet breakup requires high shear rate values, which are above what can be practically attained. For a viscosity ratio in the range of 0.1 < k < 1, the droplets are readily broken up and in this range of viscosity ratio, Ca(crit) is minimal (~0.5 to 0.6) and independent of the flow type, with the lowest Ca(crit) value obtained around k = 1. As emphasized by Janssen et al. [17,18], this does not necessarily imply that the finest morphology is always obtained at k = 1. The reason for this is that a dispersion mechanism via stepwise equilibrium breakup (which is the typical experiment underlying Figure 12) is improbable in a practical situation. For very low k values << 0.1, a log/log plot of Ca(crit) vs. (k) was fitted to a power function according to

(38) $$\mathrm{Ca}(\mathrm{crit})=0.16k^{-0.6}$$

which was in good agreement with the theoretically derived relation by Acrivos and Lo [47] from a slender body analysis:

(39) $$\mathrm{Ca}(\mathrm{crit})=0.2k^{-2/3}$$

An explanation of the highest stability of low-viscosity droplets can be given in terms of Rayleigh waves. Droplet burst can be thought of as an amplification of Rayleigh waves of a dominant wavelength. Since the dominant wavelength increases when the viscosity ratio goes down, the low-viscosity droplets are expected to exhibit highly extended shapes upon burst. As these highly extended shapes undergo high Laplace pressures (i.e., significant pressure differences across the interface) as a result of sharp ends, it can be readily explained that high shear rates are to be applied to make the shear stress exceed the Laplace pressure and to induce breakup.

According to multiple studies, deformation (especially fibrillation) of the dispersed phase in an immiscible polymer blend is facilitated by an elongational flow field and a low viscosity ratio (k ≤ 1) [36]. This explains why a majority of studies on MFCs and NFCs have been carried out using the slit-die hot stretching-quenching process.

De Bruijn [48] used experimental data to demonstrate that Ca(crit) can be fitted with the following empirical equation (for k < 4):

(40) $$\log(\mathrm{Ca}(\mathrm{crit}))=C_{1}+C_{2}\log\left(k\right)+C_{3}{\left(\log\left(k\right)\right)}^{2}+\frac{C_{4}}{\log\left(k\right)+C_{5}}$$

Here, the C_i_ values for shear and extensional flows are given in Table 1.

Common blending machines involve a mixture of elongational and shear flows and this is also true in most extruder geometries. Bentley et al. [38,49] have investigated particle deformation and breakup in various combinations of shear flow and elongational flow and experimentally demonstrated that intermediate flow types yield values of Ca(crit) in between Ca(crit)_elongation_ and Ca(crit)_shear_. Experimental data have been confirmed by numerical studies on the critical capillary number under various types of flow [46].

The above results were established for diluted Newtonian systems and the values of capillary numbers were obtained at quasi-equilibrium conditions. In commonly used blenders (extruders), the capillary number can be subdivided into different (local) capillary numbers, which vary depending on the position of the blend throughout the screws and the fluctuation of the shear rate according to the screw profile. Indeed, flow fields in an extruder are complex and there is coexistence of elongation and shear flow. Nevertheless, the flow field is much simpler in certain zones of the extruder. In the conveyor zone and the positive mixing zone, shear flow is predominant, whereas elongation flow is prevalent in the converging zone. It is also possible to generate an elongation flow by means of a stretching system at the die exit. Since the critical capillary number Ca(crit) depends on the flow type and Ca is dependent on the shear or elongational rate, the reduced capillary number Ca/Ca(crit) varies as a function of the screw profile and a variety of types of transient deformation can be found in the various extruder zones [36].

During the blending process, the length scale of the dispersed phase becomes reduced. In the initial stage of mixing, the dispersed domains are in the millimeter size range, wherefore the capillary number is significant (Ca > 1000). Consequently, the drops are deformed affinely with the matrix so as to create long liquid threads. Due to the increasing surface between the phases, the interfacial stress becomes more considerable, the capillary number goes down and the threads break up into droplets.

Depending on their size, the drops that are created may once again go through breakup. Once more, the passage of the melt through a high shear zone (compression zone) induces the stretching of the newly formed droplets into thinner fibers, and so on. Finally, an equilibrium situation is reached in which the droplets are small enough to withstand the disruptive hydrodynamic forces. Coalescence can also take place which favors the formation of new large fibrils as schematized by Luciani et al., [44]. The authors explained that the evolution of the content of fibrillar phase depends on the concentration (droplet fraction) in the medium. These results are in accordance with the findings of Favis and Chapleau [12] who discovered that when the fraction of dispersed phase is higher, there is a more pronounced generation of fibrils.

For a low concentration of the dispersed phase, when the blend is well dispersed, the average diameter observed is a result of the dynamic equilibrium in the division and coalescence phenomena governing the size of the droplets in the blend. If the content of dispersed phase increases, so does the number of particles, which enhances the collision-coalescence phenomenon among them. As a consequence, the mean diameter of the droplets increases until the largest particles are no longer stable as drops in the stress field imposed in the blender, at which time they deform into fibers. Depending on the components’ properties (rheological behavior, interfacial tension, etc) and blending conditions, there exists a composition range where both nodular and fibrillar morphologies can be seen simultaneously. A larger amount of dispersed phase increases the fiber content and reduces the droplet concentration in the blend, until phase inversion takes place. The reduction in average diameter of the nodular part as the fiber fraction increases can be related to the deformation of the larger particles into fibers. These can then coalesce with others before breaking up due to the Rayleigh instability. Fiber stability seems to be the main parameter governing whether a blend’s morphology evolves towards a pure fibrillar one (with very stable fibers) or towards a droplet-type morphology (with a high fiber division rate). For these reasons, Luciani et al., [44] introduced a novel parameter denoted q′ which depends only on the physical characteristics of the two materials involved in the blend. It is defined as:

(41) $$q^{\prime }=q\cdot B=\Gamma \Omega (k,X)/\eta_{m}$$

where ‘B’ is the initial diameter of the thread. Luciani et al. found that the lower the q′, the greater the tendency to readily obtain a stable fibrillar morphology.

Recently, Deyrail et al. [50,51] studied the in situ fiber formation in immiscible polymer blends during the crystallization (or solidification) of the dispersed phase under shear flow. They investigated the impact of the crystallization time (or quenching time for an amorphous dispersed phase), the shear rate, and the breakup time on the final morphology. The authors defined a dimensionless parameter denoted λ_DC_, which represents the ratio between the crystallization (or quenching) time and the breakup time of the filaments. If λ_DC_ >> 1, a nodular morphology is expected.

- If λ_DC_ = 1, thin fibers form nodules and thick fibers adopt a more or less pronounced wavy shape.
- If λ_DC_ << 1, a fibrillar morphology is expected.

### 4.2. Newtonian Polymer Blends: Effect of Elasticity

The above-mentioned results were obtained for Newtonian droplets and Newtonian matrix fluids. However, the non-Newtonian viscoelastic behavior of common high-molecular weight polymer blends is expected to influence the deformation and breakup of droplets in a flow field. Indeed, in the majority of immiscible polymer blends, both the drops and the surrounding medium exhibit a viscoelastic behavior so not only is the morphology of the dispersed phase determined by the viscosity force, but it is also influenced by the stress distribution around the droplets caused by elasticity force.

Several studies revealed that the viscoelasticity changes the drop deformation as well as the critical capillary number [12,20,52,53,54]. The viscoelastic drop deformation has been shown to decrease as a result of normal stresses in the drop phase (elasticity of the droplet fluid inhibiting the droplet deformation and causing the particle to break at a higher capillary number). However, according to numeorus experiments, the general behavior was that a viscoelastic matrix steadies the droplets, facilitating the deformation of particles into microfibrils (i.e., breakup occurs at a lower capillary number). Gauthier, Goldsmith and Mason [55], de Bruijn [48], Varanasi, Ryan and Stroeve [56], Ghodgaonkar and Sundararaj [57] found that the critical capillary number Ca(crit) for viscoelastic drops in a Newtonian matrix were higher than that in the corresponding Newtonian mixtures as a result of the hindering effects of drop phase normal stresses.

It is quite a complicated task to determine a quantitative relationship between viscoelasticity and droplet deformation/breakup. This is due to viscoelasticity being manifested in various ways, including first and second normal stress differences for both matrix and droplet fluids, as well as shear thinning in viscous and elastic parts of both liquids. In an attempt to better manage the contributions of fluid viscoelasticity, certain investigations have selected only one viscoelastic liquid (i.e., either the matrix or the droplet), while the other was Newtonian.

The particle behavior in binary blends where only one of the two phases is viscoelastic has been explored. Elmendorp and Maalcke [20] investigated the impact of elasticity on the breakup of isolated viscoelastic droplets in Newtonian matrices as well as of Newtonian particles in viscoelastic matrices while subjected to a simple shear flow. The authors discovered that the more elastic drops (as measured by the first normal stress difference N_1_) remained the most resistant to breakup, while the more elastic matrices gave rise to increasingly unstable drops. Milliken and Leal [21] conducted an experimental deformation/breakup study of isolated viscoelastic droplets made up of an aqueous polymer solution in a Newtonian fluid matrix subjected to a planar extensional flow generated by a four-roll-mill apparatus. They found that the viscoelastic particles deformed to a lesser extent than their Newtonian counterparts at a given capillary number and that the critical capillary number increased as compared with a Newtonian system at an equivalent viscosity ratio.

The Weissenberg number, Wi, can be used to quantiy elasticity in the droplet or matrix phase. According to Laun [58], it is the ratio of elastic to viscous forces (defined as the first normal stress difference N_1_ divided by the shear stress σ at a given deformation rate ($\dot{\gamma }$):

(42) $$\mathrm{Wi}=\frac{N_{1}}{\eta \cdot \dot{\gamma }}$$

and

(43) $$N_{1}=2\;G^{\prime }(\omega )\;{\left[1+{\frac{G^{\prime }(\omega )}{G^{\prime \prime }(\omega )}}^{2}\right]}_{\omega =\dot{\gamma }}^{0.7}$$

In the limit of low shear rate and frequency [59]:

(44) $$N_{1}(\dot{\gamma })=2G^{\prime }(\omega )$$

In similar fashion to the capillary number, the Weissenberg number increases with the shear rate, as a result of elastic forces generally growing more rapidly with the shear rate as opposed to their viscous counterparts. For a specific droplet size, there is a qualitative proportionality between Wi and Ca; however, since Ca is dependent on the particle diameter and Wi is not, these two dimensionless numbers can be independently varied by changing both the shear rate and the drop size for a specified pair of viscoelastic liquids. Since both phases can be elastic, there are two Weissenberg numbers: one for the droplet denoted Wi_d_ and one for the matrix denoted Wi_m_. Since the elastic stresses in the drop are dependent on the strength of its internal flow, which in turn depends on the viscosity ratio (particles with higher viscosity have weaker internal flows), it is clear that there generally exists a connection between the viscosity ratio and the strength of the elastic forces in the particle.

It is possible to have further control if one selects a so-called ‘‘Boger’’ fluid, which is a weakly elastic dilute polymer solution (as the viscoelastic component) in a Newtonian matrix. Boger fluids have the advantage of presenting slight or no shear thinning in the shear viscosity and ideally also in the first normal stress coefficient.

**Figure 13:**
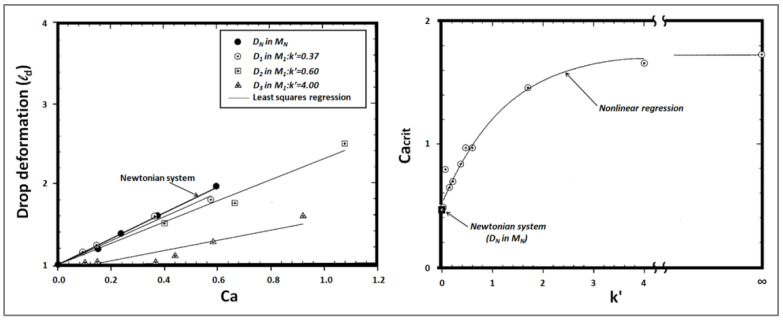
(**Left**). Drop deformation as a function of the capillary number, Ca: effect of the drop elasticity. (**Right**). Variation of the critical capillary number, Ca(crit), with the elasticity ratio k’. Adapted from reference [60].

Mighri et al. [60,61] investigated a blend of an elastic “Boger” fluid with constant viscosity as the droplet phase in a Newtonian matrix, and determined the impact of the elasticity ratio, as measured by the ratio, k’, of the Maxwell relaxation time (λe_d_) of the particle phase (also known as the characteristic elastic time),

(45) $$\lambda e_{d}=\frac{W_{i,d}}{2\dot{\gamma }}=\frac{N_{1,d}}{2\eta_{d}{\dot{\gamma }}^{2}}$$

to that of matrix phase (λe_m_) with

(46) $$\lambda e_{m}=\frac{W_{i,m}}{2\dot{\gamma }}=\frac{N_{1,m}}{2\eta_{m}{\dot{\gamma }}^{2}}$$

on the droplet deformation as well as on the critical capillary number, Ca(crit), for breakup. The authors found that there was a rise in the degree of droplet deformation and critical capillary number for breakup with an increasing elasticity ratio (Maxwell relaxation-time ratio) between the drops and the matrix, under either elongational or shear flow. This is in accordance with the aforementioned findings of Elmendorp and Berger. Under shear flow, they observed that for an elevated matrix elasticity (k’ < 0.37), the deformation of elastic droplets in an elastic matrix resembled than that of Newtonian particles in a Newtonian medium with the same viscosity ratio and interfacial tension (Figure 13a). However, for high droplet elasticity (k’ > 0.37), the elastic drops deformed less than a Newtonian droplet in a Newtonian matrix.

Furthermore, they discovered that when the elasticity ratio was low or modest, k’ (= λ_d_/λ_m_) ≤ 4, the critical capillary number Ca(crit) for droplet breakup under conditions of steady shearing became greater with increasing k’, reaching a plateau of Ca(crit) ≈ 1.75 at the high elasticity ratio (k’ ≈ 4). This can be compared with Ca(crit) ≈ 0.5 for Newtonian drops (Figure 13b). Thus, the particle’s resistance to deformation and breakup was greater with a higher elasticity ratio between the particle and the matrix phase.

Recently, single viscoelastic drops in Newtonian or viscoelastic media under simple shearing have been explored under a microscope. Lerdwijitjarud et al. [62] investigated the deformation and breakup of isolated particles of a weakly elastic liquid (Wi_d_ ≤ 0.02) in a Newtonian medium, and established that the droplet elasticity gave rise to a small increase in Ca(crit) up to 20%. The droplet’s elasticity gave rise to a reduced degree of deformation at any given shear rate and a higher critical deformation at breakup, which resulted in an increased Ca(crit). However, when the Weissenberg number was at its highest (Wi_d_ ≥ 1), this effect appeared to saturate, leading to only a modest increase in Ca(crit).

In their studies on Boger fluids, Mighri et al. [60] and Lerdwijitjarud et al. [62], observed a modest effect of the viscoelasticity on the deformation and breakup of droplets, relative to what is seen in Newtonian fluids. As an example, there was a slight change (of a factor of approximately two) in Ca(crit), whereas for highly elastic melts, very large increases of more than a decade were seen in the capillary number required for droplet breakup, see next paragraph. These studies, and those described below, suggest that large increases in Ca(crit) resulted from a new mode of drop deformation and breakup for highly elastic droplets [63].

Indeed, Vanoene [64] considered the impact of elasticity from another viewpoint. In an attempt to describe the influence of normal stresses on particle breakup for a matrix with extensional flow, an expression for the interfacial tension in flow was developed by deriving a term proportional to the difference between a second normal stress function (according to the definition in his original paper) of the particle and the matrix phase,

(47) $$\Gamma^{\mathrm{dynamIc}}=\Gamma^{\mathrm{steady}}+\frac{D}{12}\left(N_{2,d}-N_{2,m}\right)$$

where Γ^dynamic^ is the dynamic interfacial tension of a droplet of fluid d in a matrix m, Γ^steady^ is the interfacial tension of a quiescent polymer blend (in the absence of flow), D is the droplet diameter, N_2,d_ is the second normal stress difference of the dispersed phase, and N_2,m_ is the second normal stress difference of the matrix phase, which is dependent on the molecular weight, the molecular weight distribution and the shear stress. Vanoene’s results suggest that the interfacial tension under dynamic flow differed from what it would be under static flow. He demonstrated that, under dynamic flow conditions, the differences in elasticity between a blend’s components may cause the interfacial tension (known as the dynamic interfacial tension) to vary, and the obtained value could be quite different from its counterpart in the absence of flow.

Reignier et al. [65] rewrote Vanoene’s equation by replacing the second normal stress difference by the first normal stress difference giving

(48) $$\Gamma^{dynamic}=\Gamma^{\mathrm{steady}}+\frac{D}{12}\left(N_{1,d}-N_{1,m}\right)$$

which indicated that for a greater melt elasticity of the matrix as opposed to of the dispersed phase, Γ^dynamic^ should decrease as the shear increases, and inversely should become greater when the melt elasticity of the blend matrix is smaller than that of the dispersed phase.

Sundararaj et al. [59] suggested two ways to incorporate the elastic contribution: (1) by considering only the first normal stress of the drop; and (2) by taking into account the normal stresses of both droplet and matrix. In the former case, the break-up occurs when shear forces ≥ interfacial forces + droplet elasticity. When the shear rates or frequency of rotation are low, the first normal stress difference can be approximated by 2G′, where G′ is the elastic modulus, implying that the break-up condition is:

(49) $$\eta_{m}\dot{\gamma }\geq \frac{2\Gamma }{D}+2G^{\prime }{}_{d}$$

At high shear rates (>10 s^−1^), N_1_ > 2G′, but the two quantities are proportional to each other. As a first approximation to obtain the qualitative behavior, the two sides of Equation (37) are equated to obtain an expression for the drop diameter,

(50) $$D=\frac{2\Gamma }{\eta_{m}\dot{\gamma }-2G^{\prime }{}_{d}}$$

In the first case, the normal stress of the matrix was not included. However, in a polymer–polymer blend, since the matrix is also a polymer, its normal stress will also attempt to deform the drop. As a result, in the force proportionality, there exists an additional force that causes the droplet to deform and according to Ghodgaonkar et al. [57] the deformation and the final shape of the dispersed phase are the outcome of a dynamic equilibrium between the forces deforming the particle (i.e., shear stress and matrix elasticity) and forces resisting the deformation (i.e., droplet elasticity and interfacial tension). Consequently, the breakup takes place according to: shear forces + matrix elasticity ≥ interfacial forces + droplet elasticity or

(51) $$\eta_{m}\dot{\gamma }+N_{1,m}\geq \frac{2\Gamma }{D}+N_{1,d}$$

Again, the first normal stress difference N_1_ was approximated by 2G′, and by equating the two sides, the drop diameter equation becomes:

(52) $$D=\frac{2\Gamma }{\eta_{m}\dot{\gamma }-2(G^{\prime }{}_{d}-G^{\prime }{}_{m})}$$

Elastic droplets would thus resist deformation more readily at higher shear rates due to the particle elasticity having a stabilizing effect during deformation, causing the minimum attainable drop diameter to be larger when the dispersed phase is elastic.

Based on the Ghodgaonkar equation, Seo and Kim [63], introduced a new capillary number, Ca^E^, (the elastic capillary number) that they expressed as:

(53) $${\mathrm{Ca}}^{E}=\frac{\left(N_{1,m}-N_{1,d}\right)}{2\Gamma /D}$$

Dispersed droplets become deformed for Ca^E^ > 1.

These authors also explored another parameter to elucidate the effect of elasticity: the widening of a drop during deformation in immiscible viscoelastic polymer blends. The effect of elasticity on the droplet widening was first investigated by Levitt and Macosko [26] and later by Guido and Villone [66] and, based on the results obtained from these experiments, the droplet widening is inversely proportional to the ratio of the drop-to-matrix elasticities, i.e., G_r_ = G_d_/G_m_, where G_d_ and G_m_ are respectively the elastic modulus of the drop and the matrix. Based on the simple assumptions that stretching in the hoop direction is greater than that in the thickness direction and that the second normal stress difference is proportional to the first normal stress difference, Levitt and Macosko were able to derive an approximate simplified equation for the drop thickness after deformation according to:

(54) $$R_{n}{}^{\max}=\frac{\Gamma }{0.6(G_{m}-G_{d})}$$

Here, Γ is the interfacial tension and R_n_^max^ is half of the maximum thickness.

Recently, Abbassi-Sourki et al. [67] discovered that the critical capillary number can be affected by adding a compatibilizer. They expressed a new capillary number, denoted Ca(crit)^compat^, corresponding to the critical capillary number in the case where a droplet is modified by an interfacial active agent:

(55) $$\mathrm{Ca}(\mathrm{crit}){}^{\mathrm{Compat}}=\frac{\mathrm{Ca}(\mathrm{crit}){}^{\mathrm{uncompat}}}{\left[\Gamma^{\mathrm{compat}}/\Gamma^{\mathrm{uncompat}}\right]}$$

Here, Γ is the interfacial tension. The authors found that the presence of the compatibilizer causes the interfacial tension to decrease and the critical capillary number to increase.

Studies have also been performed on blend systems in which both the dispersed phase and the matrix phase are viscoelastic but without evaluating and taking into account the impact of the elasticity of the polymers in question. Wu [68] found that the critical capillary number Ca(crit) has a higher importance in the case of viscoelastic blends as compared to Newtonian ones. He also confirmed that the steady-state particle size in blends of extruded viscoelastic polymers at a viscosity ratio of unity is approximately tenfold the corresponding value for Newtonian components at an equivalent viscosity and shear rate. This investigation involved a blend of PA6,6/ethylene-propylene over a wide range of polymer viscosities in which the suspended phase of rubber particles was low (<15%).

**Figure 14:**
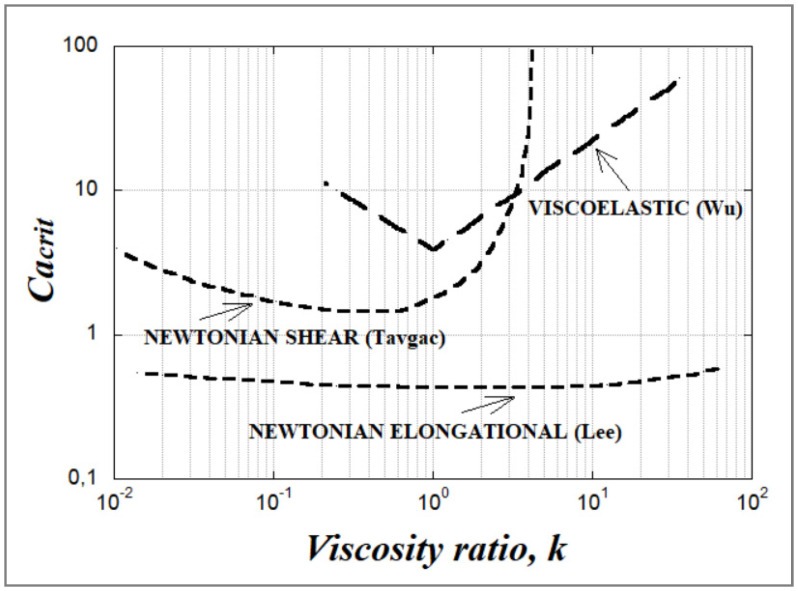
The critical capillary number as a function of the viscosity ratio. Comparison between viscoelastic systems blended in a co-rotating twin screw extruder and Newtonian liquids under steady uniform shear and in an elongational (hyperbo1ic) flow. Adapted from reference [68].

Wu [68] discovered that the variation of Ca(crit) as a function of the viscosity ratio k presented as a V-shape as opposed to the U-shape previously obtained in the case of Newtonian melts (Figure 14). He introduced an empirical equation as:

(56) $$\mathrm{Ca}(\mathrm{crit})\approx 4{\left(\frac{\eta_{d}}{\eta_{m}}\right)}^{\pm 0.84}$$

where the (+) sign in the exponent applies to viscosity ratio (k = η_d_/η_m_) values greater than unity and the (−) sign in the exponent applies to values of k below unity. He also put forward an empirical correlation relating the particle size of the suspended phase D_n_ to the viscosity ratio k for several extruded immiscible polymer blends.

Wu’s correlation is

(57) $$D_{n}=\mathrm{Ca}\cdot \frac{\Gamma }{\dot{\gamma }\eta_{m}}$$

where Γ is the interfacial tension between the two components, and $\dot{\gamma }$, is the shear rate. Here, the minimum of D_n_ corresponds to the minimum of Ca(crit) and thus to a viscosity ratio k = 1.

**Figure 15:**
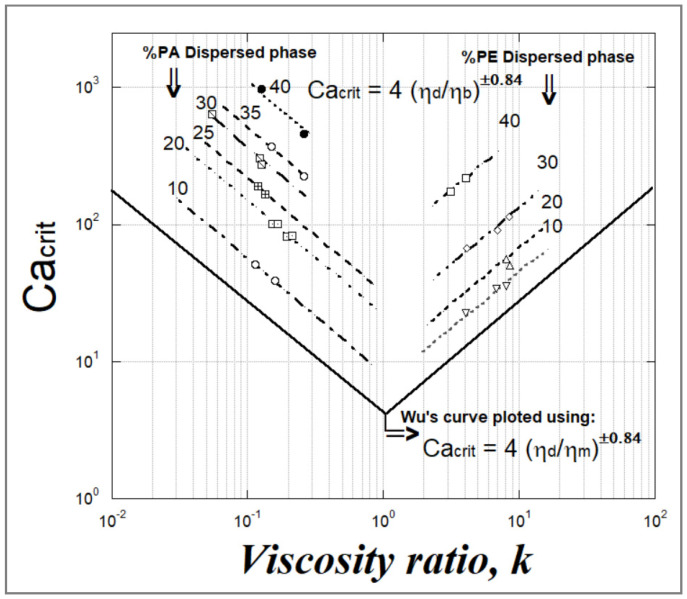
Critical capillary number vs. viscosity ratio and concentration of the dispersed phase (Φ_d_) for PE-PA6,6 blends for a wide range of mixing conditions (G: Shear rate, T: Temperature). Adapted from [69].

As Wu did not take particle coalescence into consideration, Serpe et al. [69] further developed a modification to Wu’s equation by using the viscosity of the blend $\eta_{b}$ rather than that of the matrix $\eta_{m}$ and by taking into account a term of blend composition to estimate the average drop diameter according to the empirical equations below, in which (φ_d_) and (φ_m_) are the volume fraction of respectively the suspended phase and the matrix. Serpe was able to confirm Wu’s equation for PE/PA6 blends by using this modified viscosity ratio. He demonstrated that Ca(crit) increased along with the concentration of the suspended phase (Figure 15).

(58) $$D_{n}\approx \frac{\left[\frac{4\Gamma_{1/2}}{\dot{\gamma }\;\eta_{b}}{\left(\frac{\eta_{d}}{\eta_{b}}\right)}^{\pm 0.84}\right]}{1-{\left(4\cdot \Phi_{d}\cdot \Phi_{m}\right)}^{0.8}}$$

with

(59) $$\mathrm{Ca}(\mathrm{crit})=4\cdot {\left(\frac{\eta_{d}}{\eta_{b}}\right)}^{\pm 0.84}$$

Based on the existing findings when it comes to breakup and coalescence of droplets, Fortelny et al. [70] investigated the dependence of the drop size of the minor component on its concentration in a polymer blend subjected to a simple steady shear flow. The equation they proposed takes into account both the breakup and coalescence of the particles to predict the droplet size of the minor phase. The equation is expressed

(60) $$r=r_{\mathrm{crit}}+\left(\frac{\Gamma \alpha }{\eta_{m}f_{1}}\right)\Phi$$

and here r_crit_ corresponds to the critical droplet radius as calculated from Ca(crit); α represents the probability of coalescence of the drops after collision; f_1_ is the slope of a function describing the frequency of droplet breakup at Ca(crit); and Φ is the volume fraction of the suspended phase. This relationship still contains several parameters that are not readily quantifiable for the blending of viscoelastic polymers.

However, the elasticity of the blends was not included in the empirical correlations of Wu and Serpe, even though their results were obtained from viscoelastic materials for which reason Wu’s and Serpe’s equations are not applicable for polymer blends exhibiting elasticity ratios different from those used in the studies of Wu and Serpe.

There are no quantitative relationships linking the critical capillary number to the elasticity of the droplet or matrix phase. For this reason, Lerdwijitjarud et al. [71] tried to develop quantitative dimensionless plots, similar to those that exist for Newtonian fluids, of Ca(crit) vs. other dimensionless quantities that characterize the melt viscoelasticity. However, when both the matrix and particle phases are viscoelastic, Ca(crit) is dependent on the viscosity ratio, as well as on the dimensionless elasticity of each phase. Since both viscosity and elasticity are shear-rate-dependent quantities, and since elasticity is a function of both the flow type and the flow history, both of which are complex functions of the flow in and around a particle, rigorous correlations are unlikely to be obtained.

**Figure 16:**
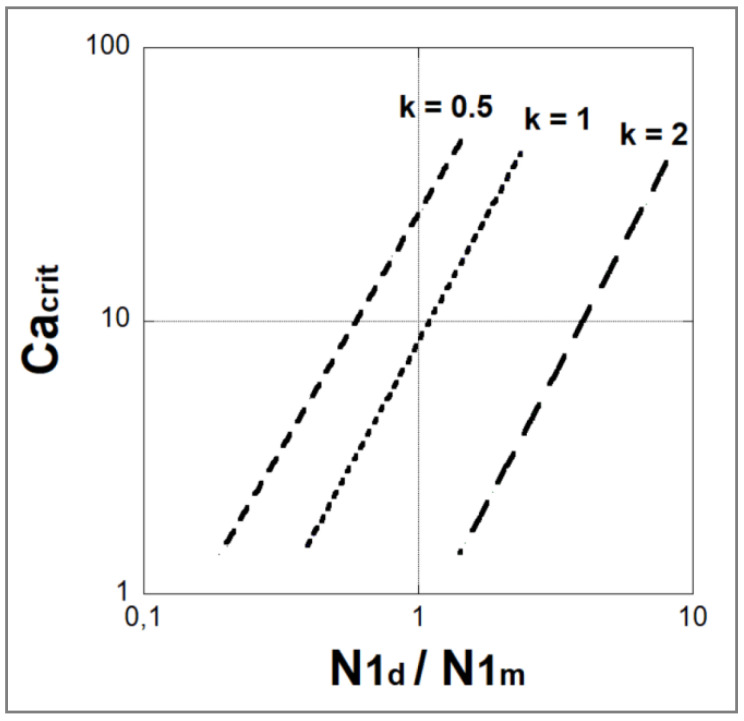
The dependence of the critical capillary number on the first normal stress difference ratio for blend systems with varying viscosity ratios. Adapted from [71].

Lerdwijitjarud et al. [71] investigated the impact of elasticity contrast, as measured by the ratio of the first normal stress differences, N_1d_/N_1m_, between the suspended phase and the medium, on the critical capillary number in uncompatibilized immiscible polymer blends under a simple shear flow. The authors found that in 80/20 *w*/*w* polyethylene/polystyrene blends sheared in a rheometer, the critical capillary numbers ranging from 2 to 30 depended on the relative magnitudes of the normal stress differences in the droplet and matrix phases as well as on the viscosity ratio (0.5, 1 and 2). These capillary numbers were between 4- and 80-fold their counterpart for breakup of a Newtonian drop in a Newtonian matrix. According to the authors, this large increase in critical capillary number (and hence droplet size) was assigned to the role of viscoelasticity. Breakup of viscoelastic particles in a viscoelastic medium is harder than for Newtonian drops in a Newtonian matrix. This is due to the contribution of both the particle elasticity and the shear-thinning of the polymer matrix. For all investigated blends, the critical capillary numbers were seen to increase with N_1d_/N_1m_, and were correlated by a power law in N_1d_/N_1m_ (Figure 16), with

(61) $$\mathrm{Ca}(\mathrm{crit})=24.6\cdot {\left(\frac{N_{1}{}_{d}}{N_{1}{}_{m}}\right)}^{1.72}\;\mathrm{for}\;a\;\mathrm{viscosity}\;\mathrm{ratio}\;k=0.5,$$

(62) $$\mathrm{Ca}(\mathrm{crit})=8.4\cdot {\left(\frac{N_{1}{}_{d}}{N_{1}{}_{m}}\right)}^{1.85}\;\mathrm{for}\;a\;\mathrm{viscosity}\;\mathrm{ratio}\;k=1,$$

(63) $$\mathrm{Ca}(\mathrm{crit})=0.7\cdot {\left(\frac{N_{1}{}_{d}}{N_{1}{}_{m}}\right)}^{1.90}\;\mathrm{for}\;a\;\mathrm{viscosity}\;\mathrm{ratio}\;k=2.$$

Experimental measurements of the normal force N_1_ are problematic and most investigations of fibrillar morphology have therefore focused on the effect of the viscosity ratio, neglecting the importance of the elasticity ratio between the dispersed and continuous phase. This has led to contradictions and ambiguity when it comes to drawing a clear parallel between the viscosity ratio and the optimal fiber formation conditions as well as temporal stability in the case of viscoelastic immiscible polymer blends. According to Min and White [72], fibrils were obtained at a viscosity ratio 0.3 < k = η_d_/η_m_ ≤ 1 for blends of undrawn melt-mixed polyethylene/polystyrene. However, Berger et al. [52] studied poly (ethylene terephthalate)/polyamide blends and found that pure shearing did not create a droplet-fiber transition when the viscosity ratio k ≤ 1. The undrawn fibrous material appeared as a dispersion of spherical particles in the polymer matrix. Fibril-in-matrix structures were only for k = 3.7 in the undrawn fibrous material. The authors confirmed that it was therefore possible to create fibril-in-matrix structures by pure shearing only when the viscosity of the dispersed phase exceeded that of the continuous one. Drawing of the fibrous material always induces fibril-in-matrix structures.

Later on, Platé et al. [73] explored different polymer pairs and indicated that good fibrillation could be achieved for a viscosity ratio in the range of 0.1 < k < 10, which was in total contradiction with Berger’s work [52]. However, the generation of a fibrillar structure by hot stretching of the polymer melt out of the slit-die (elongational flow) was easier and it was generally agreed upon that a low viscosity ratio (k ≤ 1) and stretching flow favored the fibrillation of the dispersed phase in an immiscible polymer blend [36,74]. This would explain why most investigations on MFCs have been performed using a slit-die hot-stretching quenching process rather than melt extrusion and solid state cold drawing.

The work by Lerdwijitjarud et al. [71] is of crucial importance and removes the ambiguity surrounding the conditions for obtaining a fibrillar structure in an immiscible polymer blend. The equations (48 to 50) demonstrate that in pure shear flow, in order to obtain a favorable stable fibrillar structure (low critical capillary number), the elasticity ratio should be less than unity (N_1d_/N_1m_ < 1). At the same time, the viscosity ratio should be larger than two (k ≥ 2). These results are consistent with the experimental findings of Mighri et al. [60] and Berger et al. [52].

To summarize the conclusions of the present section we may state that the critical capillary numbers (Cacrit) where all droplets will break strongly depend on the viscoelastic nature of the dispersed phase and matrix which influence the mode of deformation of droplets under a flow field. Hence, a large amount of the present section was devoted to these issues. The theoretical, empirical models and experimental investigation reports describing the drop deformation in both shear and elongational flow were examined. It was highlighted that the extension transition from drops to nanofibrils also depended on the interfacial tension Γ. On the other hand, the stability of the morphology obtained was intimately linked to the relaxation behavior and break-up kinetics of the nanofibrils occurring during NFC preparation.

## 5. Manufacturing of In Situ Nano-Fibrillar Composites (NFC)

Reinforced nanofibrillar composites (NFCs) are comprised of an isotropic polymer matrix containing nanofibrils made from a second suspended polymer and are obtained by subjecting the blend to mechanical stretching. Depending on the post processing during which the matrix polymer is processed into a continuous phase, the final structure of an NFC can exhibit both a quasi-isotropic behavior as well as varying degrees of anisotropy.

Four key requirements need to be satisfied to develop an NFC. First, the constituent polymers should have an appropriate rheological behavior in order for reinforcing nanofibrils to form; second, both of the constituent polymers must have the same processing temperature without the onset of degradation in either polymer; third, the interfacial tension between the polymer serving as the reinforcing material and the matrix has to be low enough to allow the formation of nanofibrils; and fourth, the nanofibril breakup time should be sufficiently high compared to the processing time to preserve the nanofibrils during the consolidation of the matrix [9,75]. In the thus-formed polymer blend, the minor phase could even be amorphous, in which case, the polymer blend is drawn at temperatures equal to or slightly above the glass transition temperatures (Tg) of both constituents.

There exist two standard industrial approaches for the preparation of in situ nanofibrillar composites (NFCs). The first is a “melt extrusion solid state cold drawing” process [3], in which the nanofibrils of a component with a high melting temperature are formed through “solid state cold-stretching” of the as-extruded material. The other is a “melt extrusion hot stretching-quenching” process [2], in which the nanofibrils of a component with a high melting temperature are formed through “hot-drawing” of the extrudate in the molten state and are well preserved during a solidification (or quenching) step. The major difference between the two methods is the temperature at which the minor phase is stretched to create the nanofibrils. The processing temperature during melt extrusion solid state cold drawing is below the melting temperature of each component in the system, whereas in melt extrusion, hot stretching–quenching, the system is processed at the temperature of the component with the high melting temperature.

Regardless of the process used for the preparation of the nanofibril reinforced composites, the essential stages of NFC preparation are the following:

− **Melt extrusion (mixing step)**: Melt blending followed by extrusion of the two immiscible polymers having sufficiently different melting temperatures. The matrix and/or reinforcing polymers are dried (to avoid the hydrolytic degradation) and mixed before being compounded.
− **Drawing**: The blend extrudate undergoes melt or cold drawing through roller pairs with the drawing ratio defined as the relation between the linear speeds (S_2_/S_1_) of the two sets of rollers used to draw the filament. It gives an indication of the amount of alignment imparted to the blend. The filament is then either collected on a spool or pelletized.
− **Matrix consolidation through thermal processing (isotropization step)**: The drawn filaments or pellets are injection- or compression-molded at a temperature T_proc_ above Tm of the component with the lower melting temperature and below the Tm of the higher-melting one. This converts the major phase into an isotropic matrix, while still retaining the oriented nanofibrillar structure of the component with the higher melting temperature. If T_Proc_ is too high, the nanofibrils melt and may revert to their original spherical shape, in which case the reinforcing effect might be lost.

### 5.1. Melt Extrusion and Hot-Stretching Process

**Figure 17:**
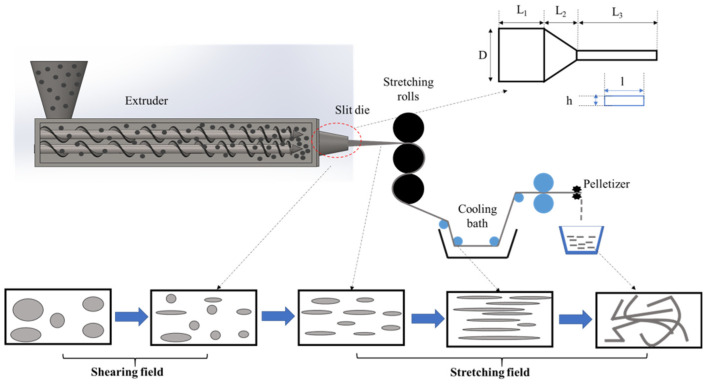
NFC manufacturing process by a melt extrusion hot-stretching-quenching process and the structural evolution of the fibrillated component through sequent steps: slit die extrusion, hot stretching, quenching, and pelletizing. Adapted from [78].

The first to report on the preparation of in situ NFCs by hot-stretching quenching were Fakirov and co-workers [3,76,77] and a schematic illustrattion of the technique is given in Figure 17, including the structural evolution of the nanofibrillated component at different processing stages. First, the two thermoplastic polymers with different melt temperatures are melt-mixed in a single-screw extruder equipped with a rectangular die (generally 10 to 30 mm wide and 1 to 3 mm thick). Then, before quenching in cold water, the material is continuously hot stretched by a take-up device with two or tree calendar rolls to form nanofibrils of the polymer with the high melting temperature in the matrix (polymer with the low melt temperature).

In the drawing zone between the die and the calendar rolls, the molten strand experiences elongational forces leading to the formation of a neck and it is in this region that the droplets are deformed to nanofibrillar structures in the molten matrix. When the elongated extrudate enters the calendar with the maximum closing pressure applied to the rolls, the thickness of the strand becomes further reduced, thereby inducing a transverse elongational component in the already drawn extrudate. At this stage the overall morphology of the ribbon adopts a highly nanolayered structure throughout, as displayed in Figure 17.

The stretching ratio (the area of the transverse section of the die to the area of the transverse section of the extrudate) is the ratio between the linear velocity of the take-up rolls (Vr) and the linear velocity of the extrudate (Ve). An apparent elongational shear rate can be defined as (Vr − Ve)/∆L, where ∆L is the length between the die and the rolls. Just after drawing, the stretched extrudate is immediately quenched in cold water (15 °C to 20 °C). This is carried out to preserve the formed nanofibrils and to avoid the risk of their breaking up via capillary instabilities. After pelletizing, liquefaction of the lower melting component (via injection or compression molding) is brought about, thus resulting in an almost complete loss of orientation of the major phase upon its solidification. However, the nanofibrils lose their orientation in order to be randomly distributed in the matrix; a step that is known as the isotropization step. It is crucial that the temperature during isotropization be kept below the T_m_ of the higher melting and already fibrillated component. In doing so, the oriented crystalline structure of the latter can be preserved, whereby the reinforcing elements of the NFC are formed.

### 5.2. Melt Extrusion and Solid-State Cold Stretching

**Figure 18:**
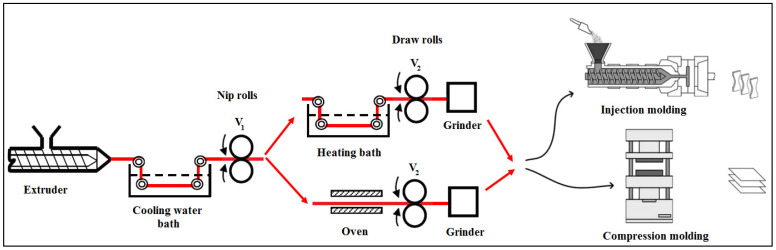
Schematic diagram of preparation process of TP/TP in situ nanofibrillar nanocomposites by a melt extrusion–solid state cold drawing process.

After exiting the capillary die of the extruder, the extrudates in the form of circular strands are quenched in a cold water bath for solidification (see Figure 18). They are then passed through a hot air oven or through a hot water bath maintained at a temperature, slightly higher than the glass transition temperature of the reinforcing component. This is followed by take-up of the strands for continuous drawing. The take-up device consists of a pair of nip rolls for which the peripheral velocity (V_1_) is maintained identical to the speed of the extrudate. Beyond the nip rolls are a pair of stretch rolls of the same diameter as the nip rolls, but whose speed (V_2_) can be varied to attain different draw ratios and, thereby reduce the cross sectional dimensions of the strands. The ratio between the stretch roll to nip roll velocities (V_2_/V_1_) corresponds to the draw (or stretching) ratio. Finally, the strands are pelletized and subsequently processed by injection or compression molding at a temperature below the T_m_ of the reinforcing component.

**Figure 19:**
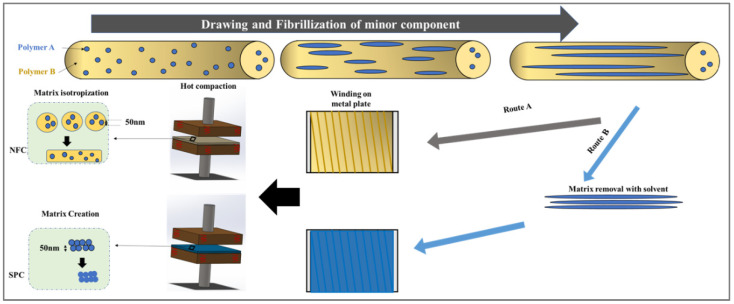
Manufacturing of nanofibrillar Polymer Polymer Composites PPCs (NFCs) (Route A) and nanofibrillar Single Polymer Composites SPCs (Route B) via the “concept of converting instead of adding”. Adapted from [4,79].

Recently, Fakirov et al. suggested a novel manufacturing route for the preparation of nanocomposites based on the concept of converting rather than adding [79]. In order to prepare nanofibrillar polymer composites (NFCs) (Figure 19, Route A), the drawn strand was wound on a metal plate and subjected to compression molding above the melting temperature of the matrix polymer A. This temperature had to be at least 40 °C below the melting temperature of the reinforcing polymer B.

To manufacture nanofibrillar single polymer composites (SPCs), one has to select Route B (Figure 19, Route B), according to which the matrix polymer A is removed from the drawn extrudates using a selective solvent. The rest of the nanofibrillar part of B is would on a metal plate and compression molded at a temperature at least 20 °C below the melting temperature of B. This way, due to a partial premelting of the surface, a small amount of isotropic matrix (binder of nanofibrils) is created. Neither technique, starting from the matrix, or from the reinforcement, include a dispersion step.

According to Fakirov et al. [79], the superior mechanical performance of the nanofibrillar polymer–polymer composites prepared according to the “concept of converting instead of adding” (Figure 19) originates from the extremely high aspect ratio of the nanofibrils, a superior adhesion to the matrix as opposed to mineral fillers and mostly from the perfect distribution of the nanofibrils in the matrix [77]. In fact, each nanofibril is individually surrounded by matrix material leaving the material without any aggregates.

The external stretching force is a prerequisite for these two methods with the different processing temperatures. As a result, additional equipment needs to be introduced to produce the stretching force. In contrast, the utilization of internal shearing and elongational forces produced during the solidification of the polymer blend melt should provide an economic and simple method to create nanofibrils in the suspended minor polymer phase when the viscosity ratio is close to or less than unity. For this reason, a dynamic packing injection molding technique was developed and employed to induce nanofibrillation by introducing a prolonged oscillatory shearing on the cooled melt blends during the solidification packing stage [80].

## 6. Morphology Development of Nanofibrillar Nanocomposites during Processing

### 6.1. Effect of Coalescence on the Morphology of NFCs Prepared by Hot-Stretching

**Figure 20:**
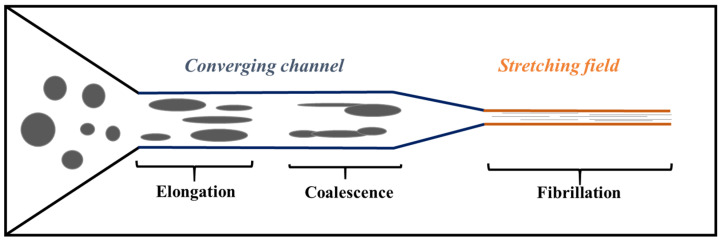
Schematic illustration of the morphological progression of in situ nanofibrillar nanocomposites prepared by a hot-stretching-quenching process [2,9].

Assuming that particles (i.e., a nodular morphology) are the predominant form of the suspended phase in a blend, the extruded melt is subjected mainly to an extensional stress in the converging cross section at the entrance of the die. In this region, the larger drops in the melt might get longer and form so-called ellipsoids, but they do not go through fibrilization. However, the intensive uniaxial extensional stress of an external drawing device causes the larger drops to easily take on the form of nanofibrils. The smaller particles, on the other hand, collide and can coalesce into larger drops in the converging transverse section of the melt, and subsequently lengthen further into nanofibrils. In short, the joint effect of coalescence and deformation results in the nanofibrilization of the particles in the suspended phase (Figure 20).

Since the size of a drop and its dispersity in the extruded melt becomes larger with the weight fraction (wf) of the suspended phase, the number of elongated nanofibrils formed both directly from the virgin droplets and from the coalesced ones increases with wf, as does the proportion of the latter in the total fiberization. This is the main reason why the dispersed phase can fiberize even at low wf (e.g., 5%) and that the number of nanofibrils increases continuously with wf [81].

Another fact in favor of the coalescence hypothesis of nanofibril formation can be found in the study by Perilla and Jana [82]. Here, the authors researched the coalescence of drops as a polymer blend (PP/PS) was extruded through a capillary die. The observed nanofibrillar morphology was attributed to the extensional components of the stress developed at the entrance of the die. However, the formed nanofibrils could either remain stable or undergo breakup, thereby generating a new population of drops. A nanofibril in simple shear flow can undergo interfacial instabilities and breakup if its radius is below a critical value, a_cr_ (in nanometers). Following the guidelines presented by Tjahjadi and Ottino [83], Perilla and Jana calculated a_cr_ at the various share rates used. It was seen that the a_cr_ values were much smaller than the radii of the nanofibrils, regardless of the share rate studied, indicating that the nanofibrils remained stable under the imposed flow conditions.

**Figure 21:**
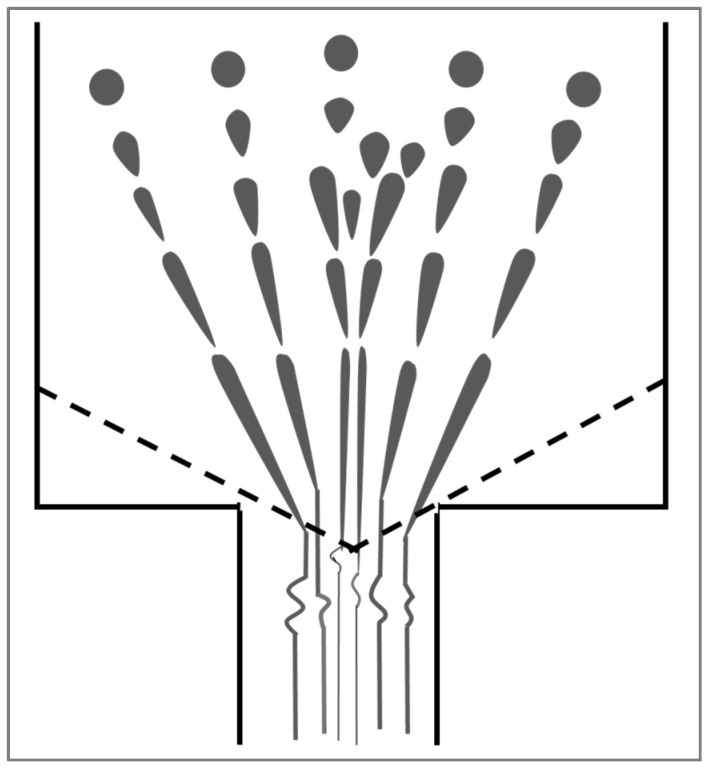
Representation of the nanofibrillation process in the entrance zone and in the die. Adapted from [84].

Other research groups have explored nanofibril formation in converging die entrances and discovered the occurrence of particle coalescence. Tsebrenko et al. studied a flow that was quenched followed by a disassembling of the capillary (16). In this case, the nanofibrils coalesced in the entrance region of the capillary and the resulting nanofibrils demonstrated a cross section with a “Vienna sausage” morphology (Figure 21).

### 6.2. Morphology Development of NFCs Prepared by Solid-State Cold Stretching

**Figure 22:**
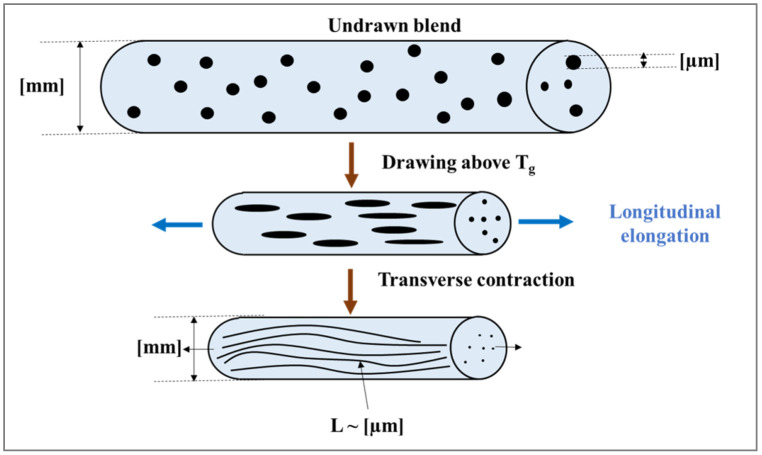
Schematic illustration of the nanofibril formation mechanism in immiscible polymer blends during cold drawing (transformation of the spherical particles into nanofibrils via coalescence under transverse contraction). Adapted from [85].

Fakirov et al. [85] proposed a qualitative mechanism of nanofibril formation during cold drawing of concentrated immiscible polymer blends (close to 50/50 wt%). This process was based on a morphological analysis using Scanning Electron Microscopy and is shown in Figure 22. The nanofibrils in the NFCs were produced by drawing the blend strand at the softening state and the authors attributed their formation to the combination of coalescence and deformation of adjacent reinforcing polymer particles. This mechanism thus suggests that, during the drawing stage above Tg of the two blend components, the dispersed particles, which initially have the form of densely populated spheres, first elongate into ellipsoids with lengths corresponding to the applied draw ratio applied. As the stretching process progresses, these ellipsoids become thinner and come into contact with each other due to their irregular movements (transverse contraction) within the matrix. This initiates end-to-end coalescence, and the particles eventually merge to form long continuous nanofibrils.

This suggested mechanism is still far from being completely understood. Indeed, one has to keep in mind that the cold drawing during the NFC preparation takes place above the glass transition, but far below the melting point of the two components. At such low temperatures, a coalescence process during the cold drawing can be almost completely excluded because of the very high viscosity (poor diffusion conditions) and the moderate flow conditions as compared to a case of molten polymers (hot stretching process). However, it is useful to mention that in the solid-state cold drawing process, the breakdown of nanofibrils is excluded because of the very high viscosity of the medium and the moderate flow conditions during the cold drawing.

## 7. Conclusions and Outlook

The concept of in situ nanofibrillar polymer–polymer composites seems to be a powerful approach to obtain polymeric materials with properties that significantly exceed those expected from common reinforced composites. This paper has highlighted the important role of rheology as a powerful tool to predict the formation and stability of nanofibrils during melt blending and post processing. Indeed, the rheological conditions to be respected for the generation of nanofibrils from polymer blends and the subsequent processing into in situ nanofibrillar NFCs have been presented. It was concluded that the combination of a viscosity ratio and an elasticity ratio below unity accompagnied with a low interfacial tension (less than 2 mN/m) seems to be favorable for the production of in situ polymer–polymer nanocomposites with a nanofibrillar morphology. On the other hand, the stability and preservation of this morphology in the NFCs depends on the breakup time of the nanofibrils, a factor that can be optimized depending on the process time used. The dispersion homogeneity of the reinforcing polymer phase, as well as the strength of the matrix/nanofibril interface, are dependent on several factors: the thermal and rheological characteristics of the neat polymers, the concentration of the dispersed phase and the processing conditions (stretching ratio and the mode of nanofibrillation under a flow field).

Applications using the nanofibrillar composite technology will most probably be extended to include novel ones with a high added value, such as fused filament fabrication 3D printing [86], gas-assisted injection molding of polymer foams [87], and forced assembly coextrusion for the development of packaging films with high permeation properties [88]. A further evolution of the NFC concept is the selective loading of nanofibrils by carbon nanotubes [89], which would give rise to a double reinforcing effect, or, in other words, to the reinforcement of a reinforced polymeric material. In addition, one obtains a new class of electroconductive nanocomposites with decent shielding properties.

Isolation of biobased nanofibrils via selective dissolution of the matrix component show potential in biomedical applications as nanofilter materials or as scaffolds for regenerative medicine or in controlled drug delivery [90]. Finally, the application of the in situ nanofibrillar blend concept shows considerable promise when it comes to the recycling of thermoplastic polymers and can be effectively utilized for the circular economy in a variety of industrial applications [4].

## Figures and Tables

**Table 1:** C_i_ coefficients for the equation Ca(crit) = f(k) [25].

	C_1_	C_2_	C_3_	C_4_	C_5_
Shear flow	−0.5060	−0.0994	0.1240	−0.1150	−0.6110
Elongational flow	−0.64853	−0.02442	0.02221	−0.00056	−0.00645

## References

[1] Wang G., Zhao G., Zhang L., Mu Y., Park C.B. (2018). Lightweight and tough nanocellular PP/PTFE nanocomposite foams with defect-free surfaces obtained using in situ nanofibrillation and nanocellular injection molding. Chem. Eng. J..

[2] Yousfi M., Soulestin J., Marcille S., Lacrampe M.-F. (2021). In-situ nano-fibrillation of poly (butylene succinate-co-adipate) in isosorbide-based polycarbonate matrix. Relationship between rheological parameters and induced morphological and mechanical properties. Polymer.

[3] Fakirov S. (2013). Nano-and microfibrillar single-polymer composites: A review. Macromol. Mater. Eng..

[4] Fakirov S. (2021). A New Approach to Plastic Recycling Via the Concept of Microfibrillar Composites. Adv. Ind. Eng. Polym. Res..

[5] Yang C., Zhang Q., Zhang W., Xia M., Yan K., Lu J., Wu G. (2021). High thermal insulation and compressive strength polypropylene microcellular foams with honeycomb structure. Polym. Degrad. Stab..

[6] Vozniak I., Hosseinnezhad R., Morawiec J., Galeski A. (2020). Microstructural Evolution of Poly (ε-Caprolactone), Its Immiscible Blend, and In Situ Generated Nanocomposites. Polymers.

[7] Shahnooshi M., Javadi A., Nazockdast H., Altstaedt V. (2020). Development of in situ nanofibrillar poly (lactic acid)/poly (butylene terephthalate) composites: Non-isothermal crystallization and crystal morphology. Eur. Polym. J..

[8] Hosseinnezhad R., Vozniak I., Morawiec J., Galeski A. (2020). Nanofibrillar green composites of polylactide/polyamide produced in situ due to shear induced crystallization. Compos. Commun..

[9] Yousfi M., Dadouche T., Chomat D., Samuel C., Soulestin J., Lacrampe M.F., Krawczak P. (2018). Development of nanofibrillar morphologies in poly(l-lactide)/poly(amide) blends: Role of the matrix elasticity and identification of the critical shear rate for the nodular/fibrillar transition. RSC Adv..

[10] Xie L., Xu H., Niu B., Ji X., Chen J., Li Z.M., Hsiao B.S., Zhong G.J. (2014). Unprecedented Access to Strong and Ductile Poly(lactic acid) by Introducing In Situ Nanofibrillar Poly(butylene succinate) for Green Packaging. Biomacromolecules.

[11] Tsebrenko M.V., Danilova G.P., Malkin A.Y. (1989). Fracture of ultrafine fibers in the flow of mixtures of non-newtonian polymer melts. J. Non-Newton. Fluid Mech..

[12] Chapleau N., Favis B.D. (1995). Droplet fiber transitions in immiscible polymer blends generated during melt processing. J. Mater. Sci..

[13] Han C.D. (1981). Multiphase Flow in Polymer Processing. Rheology.

[14] Acrivos A. (1983). The breakup of small drops and bubbles in shear flows. Ann. N. Y. Acad. Sci..

[15] Rallison J.M. (1984). The deformation of small viscous drops and bubbles in shear flows. Annu. Rev. Fluid Mech..

[16] Utracki L.A., Shi Z.H. (1992). Development of polymer blend morphology during compounding in a twin-screw extruder.1. droplet dispersion and coalescence—A review. Polym. Eng. Sci..

[17] Elemans P.H.M., Bos H.L., Janssen J.M.H., Meijer H.E.H. (1993). Transient phenomena in dispersive mixing. Chem. Eng. Sci..

[18] Janssen J.M.H., Meijer H.E.H. (1993). Droplet breakup mechanisms—Stepwise equilibrium versus transient dispersion. J. Rheol..

[19] Janssen J.M.H., Meijer H.E.H. (1995). Dynamics of liquid-liquid mixing—A 2-zone model. Polym. Eng. Sci..

[20] Elmendorp J.J., Maalcke R.J. (1985). A study on polymer blending microrheology. I. Polym. Eng. Sci..

[21] Milliken W.J., Leal L.G. (1991). Deformation and breakup of viscoelastic drops in planar extensional flows. J. Non-Newton. Fluid Mech..

[22] Rayleigh L. (1878). On the Instability of Jets. Proc. Lond. Math. Soc..

[23] Tomotika S. (1935). On the Instability of a Cylindrical Thread of a Viscous Liquid Surrounded by Another Viscous Fluid. Proc. R. Soc. A.

[24] Taylor G.I. (1932). The Viscosity of a Fluid Containing Small Drops of Another Fluid. Proc. R. Soc. London. Ser. A Contain. Pap. A Math. Phys. Character.

[25] Grace H.P. (1982). Dispersion Phenomena in High Viscosity Immiscible Fluid Systems and Application of Static Mixers as Dispersion Devices in Such Systems. Chem. Eng. Commun..

[26] Levitt L., Macosko C.W., Pearson S.D. (1996). Influence of normal stress difference on polymer drop deformation. Polym. Eng. Sci..

[27] Taylor G.I. (1934). The formation of emulsions in definable fields of flow. Proc. R. Soc. Lond. A.

[28] Rumscheidt F., Mason S. (1961). Particle motions in sheared suspensions. XII. Deformation and burst of fluid drops in shear and hyperbolic flow. J. Colloid Sci..

[29] Cox R.G. (1969). The deformation of a drop in a general time-dependent fluid flow. J. Fluid Mech..

[30] Choi S., Schowalter W. (1975). Rheological properties of nondilute suspensions of deformable particles. Phys. Fluids.

[31] Karam H., Bellinger J. (1968). Deformation and Breakup of Liquid Droplets in a Simple Shear Field. Ind. Eng. Chem. Res..

[32] Torza S., Cox R., Mason S. (1972). Particle motions in sheared suspensions XXVII. Transient and steady deformation and burst of liquid drops. J. Colloid Interface Sci..

[33] Tjahjadi M., Stone H.A., Ottino J.M. (1992). Satellite and subsatellite formation in capillary breakup. J. Fluid Mech..

[34] Utracki L. (1986). Analysis of Polymer Blends by RheologicalTechniques. J. Elastomers Plast..

[35] Meijer H.E.H., Lemstra P.J., Elemans P.H.M. (1988). Structured Polymer Blends. Makromol. Chem. Macromol. Symp..

[36] Cassagnau P., Michel A. (2001). New morphologies in immiscible polymer blends generated by a dynamic quenching process. Polymer.

[37] Stegeman Y.W., van de Vosse F.N., Meijer H.E.H. (2002). On the applicability of the Grace curve in practical mixing operations. Can. J. Chem. Eng..

[38] Stone H.A., Bentley B.J., Leal L.G. (1986). An experimental-study of transient effects in the breakup of viscous drops. J. Fluid Mech..

[39] Tomotika S. (1936). Breaking up of a Drop of Viscous Liquid Immersed in Another Viscous Fluid Which is Extending at a Uniform Rate. Proc. R. Soc. A.

[40] Kuhn W. (1953). Spontane Aufteilung von Flussigkeitszylindern in Klein Kugeln. Kolloid Z..

[41] Mikami T., Cox R., Mason S. (1975). Breakup of extending liquid threads. Int. J. Multiph. Flow.

[42] Elmendorp J.J. (1986). A study on Polymer Blending Microrheology. Delft University of Technology. Polym. Eng. Sci..

[43] Huneault M.A., Champagne M.F., Luciani A. (1996). Polymer blend mixing and dispersion in the kneading section of a twin-screw extruder. Polym. Eng. Sci..

[44] Luciani A., Jarrin J. (1996). Morphology development in immiscible polymer blends. Polym. Eng. Sci..

[45] Tavgac T.V. (1972). Drop Deformation and Breakup In Simple Shear Fields. Ph.D. Thesis.

[46] Khakhar D.V., Ottino J.M. (1986). Deformation and breakup of slender drops in linear flows. J. Fluid Mech..

[47] Acrivos A., Lo T.S. (1978). Deformation and breakup of a single slender drop in an extensional flow. J. Fluid Mech..

[48] De Bruijn R.A. (1991). Deformation and Breakup of Drops In Simple Shear Flows. Ph.D. Thesis.

[49] Bentley B.J., Leal L.G. (1986). An experimental investigation of drop deformation and breakup in steady, two-dimensional linear flows. J. Fluid Mech..

[50] Deyrail Y., Fulchiron R., Cassagnau P. (2002). Morphology development in immiscible polymer blends during crystallization of the dispersed phase under shear flow. Polymer.

[51] Deyrail Y., Michel A., Cassagnau P. (2002). Morphology in immiscible polymer blends during solidification of an amorphous dispersed phase under shearing. Can. J. Chem. Eng..

[52] Berger W., Kammer H.W., Kummerlőwe C. (1984). Melt rheology and morphology of polymer blends. Die Makromol. Chem..

[53] Tsebrenko M.V., Rezanova N.M., Tsebrenko I.A. (1999). Fiber-forming properties of polymer mixture melts and properties of fibers on their basis. Polym. Eng. Sci..

[54] Pesneau I., Kadi A.A., Bousmina M., Cassagnau P.H., Michel A. (2002). From polymer blends to in situ polymer/polymer composites: Morphology control and mechanical properties. Polym. Eng. Sci..

[55] Gauthier F., Goldsmith H.L., Mason S.G. (1971). Particle motions in non-Newtonian media. Rheol. Acta.

[56] Varanasi P.P., Ryan M.E., Stroeve P. (1994). Experimental-study on the breakup of model viscoelastic drops in uniform shear-flow. Ind. Eng. Chem. Res..

[57] Ghodgaonkar P.G., Sundararaj U. (1996). Prediction of dispersed phase drop diameter in polymer blends: The effect of elasticity. Polym. Eng. Sci..

[58] Laun H.M. (1986). Prediction of elastic strains of polymer melts in shear and elongation. J. Rheol..

[59] Sundararaj U., Macosko C.W. (1995). Drop breakup and coalescence in polymer blends—The effects of concentration and compatibilization. Macromolecules.

[60] Mighri F., Carreau P.J., Ajji A. (1998). Influence of elastic properties on drop deformation and breakup in shear flow. J. Rheol..

[61] Mighri F., Ajji A., Carreau P.J. (1997). Influence of elastic properties on drop deformation in elongational flow. J. Rheol..

[62] Lerdwijitjarud W., Larson R.G., Sirivat A., Solomon M.J. (2003). Influence of weak elasticity of dispersed phase on droplet behavior in sheared polybutadiene/poly(dimethyl siloxane) blends. J. Rheol..

[63] Seo Y., Kim J. (2001). Structure development of TLCP ternary blends during biaxial elongational flow. Polymer.

[64] Vanoene H. (1972). Modes of dispersion of viscoelastic fluids in flow. J. Colloid Interface Sci..

[65] Reignier J., Favis B.D., Heuzey M.C. (2003). Factors influencing encapsulation behavior in composite droplet-type polymer blends. Polymer.

[66] Guido S., Villone M. (1998). Three-dimensional shape of a drop under simple shear flow. J. Rheol..

[67] Abbassi-Sourki F., Huneault M.A., Bousmina M. (2009). Effect of compatibilization on the deformation and breakup of drops in step-wise increasing shear flow. Polymer.

[68] Wu S.H. (1987). Formation of dispersed phase in incompatible polymer blends—Interfacial and rheological effects. Polym. Eng. Sci..

[69] Serpe G., Jarrin J., Dawans F. (1990). Morphology-processing relationships in polyethylene-polyamide blends. Polym. Eng. Sci..

[70] Fortelny I., Kovar J. (1989). Droplet size of the minor component in the mixing of melts of immiscible polymers. Eur. Polym. J..

[71] Lerdwijitjarud W., Sirivat A., Larson R.G. (2002). Influence of elasticity on dispersed-phase droplet size in immiscible polymer blends in simple shearing flow. Polym. Eng. Sci..

[72] Min K., White J.L., Fellers J.F. (1984). Development of phase morphology in incompatible polymer blends during mixing and its variation in extrusion. Polym. Eng. Sci..

[73] Plate N.A., Kulichikhin V.G., Talroze R.V. (1991). Mesophase polymers in the coming decade—Problems and trends. Pure Appl. Chem..

[74] Dreval V.E., Vinogradov G.V., Plotnikova E.P., Zabugina M.P., Krasnikova N.P., Kotova E.V., Pelzbauer Z. (1983). Deformation of melts of mixtures of incompatible polymers in a uniform shear field and the process of their fibrillation. Rheol. Acta.

[75] Chomat D., Soulestin J., Lacrampe M.F., Sclavons M., Krawczak P. (2015). In situ fibrillation of polypropylene/polyamide 6 blends: Effect of organoclay addition. J. Appl. Polym. Sci..

[76] Fakirov S., Duhovic M., Maitrot P., Bhattacharyya D. (2010). From PET nanofibrils to nanofibrillar single-polymer composites. Macromol. Mater. Eng..

[77] Fakirov S., Bhattacharyya D., Shields R. (2008). Nanofibril reinforced composites from polymer blends. Colloids Surf. A Physicochem. Eng. Asp..

[78] Wang K., Chen F., Li Z., Fu Q. (2014). Control of the hierarchical structure of polymer articles via “structuring” processing. Prog. Polym. Sci..

[79] Fakirov S. (2018). Nanofibrillar polymer–polymer and single polymer composites via the “converting instead of adding” concept–examples of true polymer nanocomposite. Adv. Ind. Eng. Polym. Res..

[80] Su R., Jiang K., Ge Y., Hu S., Li Z., Li X., Wang K., Zhang Q., Fu Q., Yang F. (2011). Shear-induced fibrillation and resultant mechanical properties of injection-molded polyamide 1010/isotactic polypropylene blends. Polym. Int..

[81] Zhao C., Mark L.H., Alshrah M., Soltani I., Lee P.C., Park C.B. (2019). Challenge in manufacturing nanofibril composites with low matrix viscosity: Effects of matrix viscosity and fibril content. Eur. Polym. J..

[82] Perilla J.E., Jana S.C. (2004). A time-scale approach for analysis of coalescence in processing flows. Polym. Eng. Sci..

[83] Tjahjadi M., Ottino J.M. (1991). Stretching and breakup of droplets in chaotic flows. J. Fluid Mech..

[84] Tsebrenko M.V., Yudin A.V., Ablazova T.I., Vinogradov G.V. (1976). Mechanism of fibrillation in flow of molten polymer mixtures. Polymer.

[85] Fakirov S., Bhattacharyya D., Lin R., Fuchs C., Friedrich K. (2007). Contribution of coalescence to microfibril formation in polymer blends during cold drawing. J. Macromol. Sci. Part B Phys..

[86] Jeantet L., Regazzi A., Taguet A., Pucci M.F., Caro A.-S., Quantin J.-C. (2021). Biopolymer blends for mechanical property gradient 3D printed parts. Express Polym. Lett..

[87] Zhao C., Mark L.H., Kim S., Chang E., Park C.B., Lee P.C. (2021). Recent progress in micro-/nano-fibrillar reinforced polymeric composite foams. Polym. Eng. Sci..

[88] Embabi M., Kweon M.S., Chen Z., Lee P.C. (2020). Tunable Tensile Properties of Polypropylene and Polyethylene Terephthalate Fibrillar Blends through Micro-/Nanolayered Extrusion Technology. Polymers.

[89] Fakirov S., Rahman M.Z., Pötschke P., Bhattacharyya D. (2014). Single Polymer Composites of Poly (B utylene Terephthalate) Microfibrils Loaded with Carbon Nanotubes Exhibiting Electrical Conductivity and Improved Mechanical Properties. Macromol. Mater. Eng..

[90] Mishra R. (2018). Efficient In-Situ Reinforced Micro/Nano Fibrillar Polymer-Polymer Composites: A New Class of Materials for Biomedical Application. J. Med. Chem. Drug Des..

